# Variability in Preprocessing and Analysis of the Error‐Related Negativity in Adult Response‐Conflict Studies: A Systematic Review

**DOI:** 10.1111/psyp.70402

**Published:** 2026-10-08

**Authors:** Katharina Paul, Johanna Paping, Jan Wacker

**Affiliations:** ^1^ Department of Differential Psychology and Psychological Assessment Institute of Psychology, University of Hamburg Hamburg Germany; ^2^ Aerospace Psychology Institute of Aerospace Medicine, German Aerospace Center Cologne North Rhine‐Westphalia Germany

**Keywords:** analytic flexibility, electroencephalography, error‐related negativity (ERN), preprocessing, preregistration, reproducibility

## Abstract

The analysis of EEG (electroencephalography) data affords many defensible choices, creating substantial researcher degrees of freedom. While stimulus‐locked components such as the N400 show wide methodological variability and incomplete reporting, it has been unclear whether this generalizes to the response‐locked error‐related negativity (ERN). We conducted a PRISMA‐guided systematic review of ERN studies published 1995–2025. From a chronologically stratified sample of 202 studies of adults using conventional response‐conflict tasks and conventional ERP analyses, we extracted preprocessing and quantification choices, assessed reporting completeness, examined temporal trends, and tested predictors of pipeline decisions (e.g., montage size, amplifier, analysis software, task). ERN pipelines and order of analysis steps were highly heterogeneous, no two studies used the same workflow, and reporting gaps persisted. Several analytic decisions showed modest temporal shifts, yet overall diversity remained high. Sample sizes increased over time but remained insufficient for typical small‐to‐moderate sized effects, and detailed preregistrations were extremely rare. None of the 32 processing and analysis choices examined in more detail varied systematically by task, and only a subset was linked to recording parameters (e.g., montage size, amplifier, analysis software). Across adult ERN studies using conventional response‐conflict tasks and conventional ERP analyses, we observed substantial methodological heterogeneity, limited convergence in analytic practice, and considerable potential for analytic flexibility. Together this may complicate the comparison and synthesis of findings across studies.

## Impact Statement

This systematic review shows that preprocessing and analysis pipelines for the error‐related negativity (ERN) vary widely across studies published between 1995 and 2025. This heterogeneity is only weakly explained by task or recording setup and indicates substantial potential for analytic flexibility. By mapping where variability concentrates, the review provides a basis for more informed interpretation of ERN results, targeted multiverse analyses, and the development of context‐sensitive methodological guidance for ERN research.

Complex EEG preprocessing and analysis workflows give researchers an almost unlimited menu of defensible choices, creating what has been called a “multiverse” of alternative pipelines (e.g., Clayson 2024; Luck and Gaspelin 2017; Paul et al. 2022, 2025; Simmons et al. 2011). Investigating these alternative pipelines through multiverse (Steegen et al. 2016) or forking‐path analyses (Wacker 2017) has shown that equally reasonable pipelines applied to the same EEG data can yield substantially varying final results (e.g., Clayson et al. 2021; Šoškić et al. 2024). Our own work has even demonstrated that pipelines can yield both theoretically predicted and opposite patterns of results (e.g., Beauducel et al. 2024; Paul et al. 2025). In the absence of detailed preregistrations or alternative approaches to constrain researcher degrees of freedom, each analytic decision introduces additional flexibility that may influence the resulting statistical conclusions. Pipeline flexibility has therefore been identified as a potential source of variability in reported findings. This combination of analytical flexibility and the chronically low statistical power typical of many EEG studies (e.g., Clayson et al. 2019; Larson and Carbine 2017), markedly inflates the probability of spurious but statistically significant effects. Together, all of this may contribute significantly to the questionable replicability of some findings reported in the ERP literature (Clayson, Carbine, et al. 2025; Pavlov et al. 2021).

Whereas methodological variability is not inherently problematic, as long as the field converges on somewhat clear best‐practice standards or (situation dependent) decision guidelines, current evidence suggests that such consensus is still elusive. In their review, Šoškić et al. (2022) audited 132 published studies of the N400 event‐related potential (ERP) and found considerable methodological variability, only a small fraction of which could be attributed to trends over time like shifts toward using mastoids as offline reference, narrower bandpass filter options, a combination of several artifact correction methods, and longer baseline windows. In the only other systematic review, Jacobsen et al. (2025) reported similar heterogeneity in 27 mobile EEG studies of the P300 component, noting that every study used a unique preprocessing pathway. Due to the small number of studies, they could not examine time trends. Thus, substantial variability persists even within specific ERP domains, and stimulus‐locked components are the only ones scrutinized so far. Whether comparable heterogeneity, or any directional change over the past three decades, can be found for other ERPs remains unknown, leaving a critical gap in our understanding of pipeline‐related researcher degrees of freedom.

To address this gap, we focus on the error‐related negativity or error‐negativity (ERN or Ne). The ERN is a negative‐going deflection in the ERP implicated in action monitoring and error detection that occurs around 50–150 ms after an erroneous response in a speeded reaction time task (Gehring et al. 1990; Hohnsbein et al. 1989). The ERN is ideally suited for a variability audit for several reasons. To begin, it is response‐locked, providing a principled contrast to the stimulus‐locked N400 and P300 previously already surveyed (Jacobsen et al. 2025; Šoškić et al. 2022). Moreover, more than three decades of sustained interest have produced a large and chronologically broad literature (Gehring et al. 2018). Experimental paradigms used to elicit the ERN are comparatively limited in number and variability (compared, for instance, to the P300), reducing paradigm‐driven heterogeneity. Importantly, pipeline variability (in reference scheme, filter settings, baseline or electrode selection) has been shown to influence ERN amplitude, effects associated with it (e.g., of gender), and its reliability (e.g., Clayson et al. 2021; Klawohn et al. 2020). Systematically mapping that variability will therefore illuminate how researcher degrees of freedom operate in one of the field's most widely studied response‐locked ERPs.

Against this backdrop, the present systematic review pursues four tightly linked aims: First, we describe and quantify the between‐study heterogeneity of preprocessing and quantification pipelines used for the ERN, testing if the heterogeneity reported by Šoškić et al. (2022), and Jacobsen et al. (2025) generalizes to this response‐locked ERP. A detailed documentation of preprocessing/analysis pipelines in the more recent literature will also provide a basis to assess representativeness of future multiverse analyses and studies systematically comparing pipelines submitted by different groups of participating researchers for the same data set (i.e., many‐analyst approaches, e.g., Trübutschek et al. 2024).

Second, we chart how pipeline choices have evolved across the 1995–2025 period by asking whether particular analytic decisions such as reference choice, filter bandwidth, or baseline interval show a systematic drift, and whether the overall preprocessing/analysis variability has narrowed, remained stable, or widened with time. A systematic drift could signal emerging best‐practice norms and/or that standardization efforts through guidelines have taken hold (Picton et al. 2000; Keil et al. 2014; Kappenman and Luck 2016). Whereas continued scatter or even increased variability would imply that consensus is still distant, possibly because of methodological innovation. Increased variability would further highlight the necessity of approaches to reduce researcher degrees of freedom like preregistration (Clayson, Mcdonald, et al. 2025; Paul et al. 2021) or blind analysis (MacCoun and Perlmutter 2015).

Third, we evaluate whether observable features of the recording setup (e.g., number of EEG channels, presence of dedicated EOG channels, amplifier, preprocessing software, experimental task, sample size) predict analytical decisions. For instance, use of an average reference is only justified for montages with a larger number of electrodes (Junghöfer et al. 1999), ocular correction algorithms differ in their reliance on EOG channels (e.g., Gratton et al. 1983), and amplifier systems can influence data quality and therefore data preprocessing. Identifying such equipment‐driven choices would help laboratories benchmark their own pipelines against comparable setups. Conversely, this will guide future multiverse analyses based on their particular recording setups (e.g., Clayson et al. 2021; Klawohn et al. 2020; Paul et al. 2025; Šoškić et al. 2024) toward defensible comparisons (Del Giudice and Gangestad 2021) and ensure computational feasibility.

Finally, we track changes in sample size and the proportion of preregistered studies, two indicators of statistical power and analytic transparency that feature prominently in current reproducibility debates (Clayson, Carnine, et al. 2025; Del Giudice and Gangestad 2021; MacCoun and Perlmutter 2015; Paul et al. 2021). Together, these objectives will reveal whether the ERN literature is converging on shared methodological standards or whether substantial methodological flexibility remains present in the literature.

To address these aims we carried out a PRISMA‐guided systematic review (Page et al. 2021), screening the ERN literature and extracting a chronologically stratified corpus of primary studies. The next section details the search strategy, screening flow, coding scheme, and statistical procedures used to analyze pipeline choices, temporal patterns, equipment‐based moderators, and indicators of power and transparency.

## 1 Methodology

Where applicable, this systematic review adhered to the Preferred Reporting Items for Systematic Reviews and Meta‐Analyses (PRISMA) guidelines (Page et al. 2021).

### 1.1 Database Search

A systematic literature search was conducted in PubMed, and PsycINFO using the search terms [(ERN OR Error‐Related Negativity OR Ne) AND (EEG OR ERP)]. The database searches were conducted using the platforms' default settings. In PubMed, this included keyword searches across all fields along with automatically integrated MeSH (Medical Subject Headings) terms, in the PsycINFO the database‐default field mappings were used. The search was limited to articles published after 1995 and was conducted first in November 2022, yielding 3391 unique results. It was repeated January 2026 yielding 518 additional results. Additionally, four manuscripts listed in the review from Saunders and Inzlicht (2020) but missing in our search results were added, yielding 3913 records.

### 1.2 Paper Selection and Screening

Inclusion and exclusion criteria were refined during the screening process. First, publications were excluded if they were not published in English or if they were not peer‐reviewed journal articles reporting original research (this included conference abstracts due to missing method section, reviews, meta‐analyses, comparison/development of analysis pipelines/steps). Studies were excluded if the population included individuals with neurological conditions such as Alzheimer's disease or epilepsy, as these conditions could affect EEG signals. Research utilizing experimental tasks outside of standard response‐conflict paradigms, such as the Flanker, Stroop, Go/No‐Go, or Simon tasks, was excluded. Additionally, studies incorporating Virtual Reality, MRI, or brain stimulation were not considered, as these methods could alter data preprocessing in particular ways. Papers relying solely on advanced analysis techniques, such as Independent Component Analysis (ICA) or Source Localization, without a conventional ERP analysis, were removed. To exclude likely systematic differences related to age, only adult samples (18–60 years) were included, thereby excluding studies with children and older adults. Based on title, abstract, and full‐text screening, 3367 papers were excluded. The remaining 546 entries were sorted by publication year and first author. To create a subsample, every third paper from this list was selected, stratified by year, yielding a final sample of 202 papers. The PRISMA flow diagram summarizing the screening procedure can be seen in Figure 1.

**FIGURE 1:**
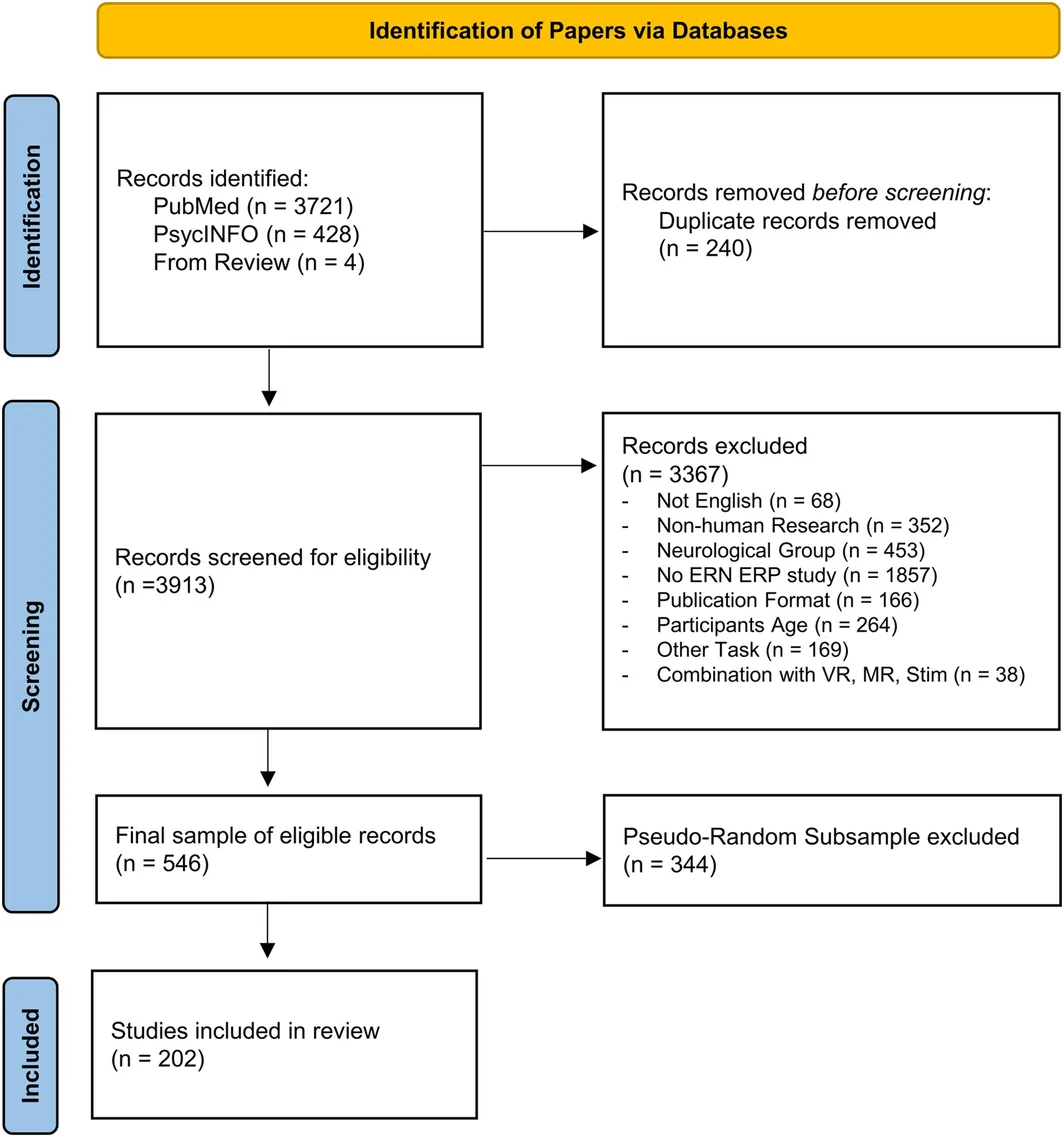
PRISMA Flow Diagram of the Screening Procedure.

### 1.3 Data Extraction

For each eligible paper, methodological details and publication metadata were systematically extracted, see below/supplement. The selection of extracted variables was guided by Šoškić et al. (2022). A total of 60 variables were documented, categorized into broader methodological topics, including experimental designs, equipment specifications, data recording procedures, preprocessing steps, and ERP quantification methods. A complete list can be found in the Supplement. Key variables relevant for explaining variance in preprocessing and quantification practices are summarized below.

In order to reduce the complexity of the data and focus on key patterns, the collected categorical choices were grouped based on methodological similarity. Continuous variables were categorized into bins based on visual inspection of their histograms, and bin boundaries were chosen to align with apparent breaks or deviations in the frequency distribution. A detailed description of the combined choices and the histograms can be found in the Figure S1. The category “Not reported” was generally retained as an explicit category because reporting practices themselves constitute an important aspect of methodological heterogeneity in the ERP literature. The category “Other” was included to avoid highly sparse category structures. Category definitions are listed below and frequencies are provided in Figure 3 and Table S6.

*Experimental Design*: Sample size, within or between subject design, experimental task (Flanker, Stroop, Go/No‐Go, Simon or another stimulus–response task).

*Equipment Specifications*: Amplifier type (Biosemi, BrainProducts, Neuroscan, Geodesic, Other), number of recorded channels (3–15, 19–34, 36–72, 128–256), recording of dedicated EOG channels (yes/no), electrode type (active/passive), preprocessing software (BVA, EEGLAB, Scan, BESA, other).

*Preprocessing*: Sampling Rate during Analysis (closest to 125 Hz, 250 Hz, 500 Hz, 1000 Hz, 2000+ Hz), reference of data (Common Average, mastoids, CSD, earlobes, other), low‐pass filtering (10–25 Hz, 30 Hz, 35–80 Hz, 100–300, not applied/reported), high‐pass filtering (0.01–0.07 Hz, 0.1 Hz, 0.15–0.75 Hz, 1–2 Hz, not applied/reported), screening for bad channels (yes/no), correction for ocular artifacts (regression with EOGs, ICA/BSS methods, no specific correction but general artifact removal, not applied), correction for other artifacts (automatic (e.g., based on absolute amplitude, voltage difference, bad channels), visual screening, combination of automatic and manual approaches, not applied/reported).

*ERN Quantification*: Length of baseline (10–50 ms, 100 ms, 150 ms, 200 ms, 300–600 ms), center of the baseline window (−700 to −450 ms, −400 to −200 ms, −175 to −50 ms, −25 to −5 ms), length of the ERN time window (20–50 ms, 56–90 ms, 100 ms, 104–200 ms, 230–300 ms), center of ERN's time window (0–45 ms, 50 ms, 52–90, 100–150 ms), central electrode (FCz, Fz, Cz, FCz&Cz, FCz&Fz, Fz&Cz, not relevant (e.g., for topographical analyses)), number of fronto/central electrodes (1, 2–3, 4–7, 8–12, 15–21, not relevant), averaging across relevant electrodes (pooled, separately, only one, not relevant), amplitude detection (mean, peak, localized mean (mean around subject's peak), component‐to‐component, not relevant).

Data extraction until 2022 was carried out by one researcher (J.P.), and completed through 2025 by a second researcher (KP). KP reviewed the extracted data and clarified ambiguities, and finalized categories (primarily for Artifact Correction Methods) and corrected and double‐checked unusual entries (e.g., not reported/not applied). An independent reviewer (JW) extracted 25 relevant variables in a subset of 44 studies (21%). Interrater agreement between the corrected data and JW's extraction was calculated using Cohen's κ (two raters, unweighted). *κ* values ranged from 0.73 to 1.00 (*M* = 0.91), indicating substantial to excellent agreement. Variable‐level κ coefficients are reported in Table S3.

### 1.4 Analysis

#### 1.4.1 Descriptives

We report both the distribution of methodological choices and their variability. To examine the diversity of choices, entropy was calculated using the Shannon entropy formula and normalized to range from 0 to 1, where 1 indicates the highest uncertainty/diversity:

$$H\left(X\right)=-\frac{\sum_{i=1}^{k}P\left(c_{i}\right)\log\left(P\left(c_{i}\right)\right)}{\log\left(k\right)}$$

With $P\left(c_{i}\right)$ as the probability of the $i^{\mathrm{th}}$ choice, and k as the total number of choices for that step.

In addition to overall entropy, we examined how much variability could be explained by other methodological choices (e.g., how the entropy of filter choices changes when controlling for the number of recorded channels). We applied an entropy decomposition method that separates total variability into between‐group (explained) and within‐group (residual) components, based on weighted group‐level entropy. This approach allowed us to estimate the proportion of variability attributable to structured methodological differences versus residual methodological heterogeneity.

#### 1.4.2 Explaining Variability

In order to explain the variability in choices, we tested several predictors including year, sample size, experimental task, amplifier model, electrode types, number of recorded channels, recording of dedicated EOG channels, and the software used to analyze the data. We tested if these variables explain variability in either the preprocessing of the EEG data (reference, filtering, artifact detection method, ocular correction method, scanning for bad channels) or the quantification of the ERP component (baseline, ERN time window, electrodes, amplitude detection method). All associations were examined in separate models.

Analyses were chosen based on the scale of the dependent variable. Binary outcomes (e.g., whether bad channels were detected) were analyzed using binary logistic regression, whereas nominal outcomes with more than two categories were analyzed using multinomial logistic regression. Continuous methodological variables were also represented using the methodologically interpretable categories described above and analyzed according to the number of outcome categories. When both the predictor and outcome were continuous (e.g., publication year and filter frequency), we additionally fitted linear regression models. For these linear‐regression analyses, extreme values exceeding 10 times the interquartile range were removed, and a rankit transformation was applied to approximate normality. Predictor effects in binary and multinomial models were evaluated using likelihood‐ratio χ^2^ tests. Model fit was quantified using McFadden's pseudo‐*R* ^*2*^, with 95% confidence intervals estimated using percentile bootstrapping. For brevity, McFadden's pseudo‐ *R* ^*2*^ is denoted as *R* ^*2*^ for all logistic and multinomial models throughout the Results and tables. For linear regression models, *R* ^*2*^ refers to the conventional coefficient of determination; its 95% confidence intervals were likewise estimated using percentile bootstrapping.

To illustrate the cell‐level patterns underlying selected associations, mosaic plots were supplemented with standardized Pearson residuals calculated from the corresponding contingency tables. Cells with residuals ≥ 2 or ≤ −2 were marked as overrepresented (+) or underrepresented (−), respectively. Only cells containing at least five observations were displayed.

To limit the interpretation of potentially trivial associations given the large number of analyses, findings were highlighted only when they met both a stringent significance threshold of (*p* < 0.001) and an *R* ^2^ threshold of at least 0.10.^1^ Nevertheless, given the broad scope of the present investigation and the large number of evaluated associations, the analyses should be interpreted as exploratory and descriptive in nature.

#### 1.4.3 Analysis Order

To examine variability in preprocessing step ordering, we encoded five preprocessing steps (Filtering, Referencing, Epoching, Artifact Detection, Blink Removal) for each paper's pipeline (allowing for steps to be repeated or omitted). Pipeline variability was quantified using three complementary measures. First, we computed all pairwise Levenshtein distances between pipeline strings and report the mean and standard deviation as a measure of overall ordering variability. The Levenshtein distance reflects the minimum number of insertions, deletions, or substitutions required to transform one sequence into another. Second, we counted the number of unique pipeline orderings as a proportion of all papers. Third, we calculated normalized entropy over the distribution of observed pipeline orders (when represented as a string).

#### 1.4.4 Use of AI‐Assisted Tools

During the preparation of this manuscript, the authors used ChatGPT (OpenAI) to assist with the development and debugging of R code and with language editing and refinement of selected passages. All AI‐assisted code was reviewed, tested, and validated by the authors before use. The tool was not used for data generation, data extraction, or autonomous interpretation of the results. The authors reviewed and edited all outputs and take full responsibility for the analyses and content of the manuscript.

## 2 Results

### 2.1 Sample Characteristics

The final sample included studies from 171 different first authors, spanning across 9 different countries, of which the United States accounted for most (36.63%), followed by Germany (17.33%). Most studies were published in recent years (e.g., 26.24% between 2016 to 2020 in comparison to 3.96% between 1995 to 2000).

The included studies applied five distinct reaction‐choice tasks. The Flanker task was the most commonly used (63.37%), followed by the Go/No‐Go task (13.37%). Around 12% of studies utilized alternative or custom tasks, including four‐choice letter tasks, hybrid designs combining conventional tasks (e.g., Flanker and Go/No‐Go), between‐hand choice reaction time tasks, and three‐choice response tasks.

Regarding participant age, the mean age across all studies was 26.23 years (SD = 7.69) ranging from 18 to 60. The variability in sample age was relatively narrow, with an average within‐study standard deviation of 5.14 years (SD = 3.54) ranging from 1 to 14.

### 2.2 Reporting Fidelity

Regarding the completeness of reporting, we found that the information most frequently missing was about the Processing Software (39.11%) and the Amplifier Model (17.82%). In contrast, information most frequently mentioned included the number of recorded channels (0.50% missing), sampling rate (2.48% missing), or the reference (1.49% missing). Regarding the quantification of the ERN, the electrodes where the ERN was measured were always mentioned where relevant (0% missing) and the specific method (e.g., peak or mean) was described by most studies (2.97% missing). In comparison, the time window in which the ERN was defined was missing in 7.43% of the studies. For other preprocessing steps, it remained unclear whether they were not applied or not reported (filtering, artifact correction), but 13.37% of studies did not mention any form of correction/exclusion for ocular or other artifacts.

### 2.3 Sample Size Development

Sample sizes were larger for between‐subjects than within‐subject effects (*M*_Between_ = 44.00, SD = 65.59 vs. *M*_Within_ = 20.00, SD = 39.24). Importantly, there was also a trend toward larger samples over time, see Figure 2. For example, an average sample size of *M*_Between_ = 31.70 (SD = 18.96) for years 2000–2005 vs. *M*_Between_ = 114.54 (SD = 106.82) for years 2021–2025. This relationship was significant over all studies, as well as separately for within‐ and between‐subjects designs (*t* (> 66) > 5.75, *p* ≤ 0.001, *R* ^*2*^ > 0.24 [0.11–0.39]).

**FIGURE 2:**
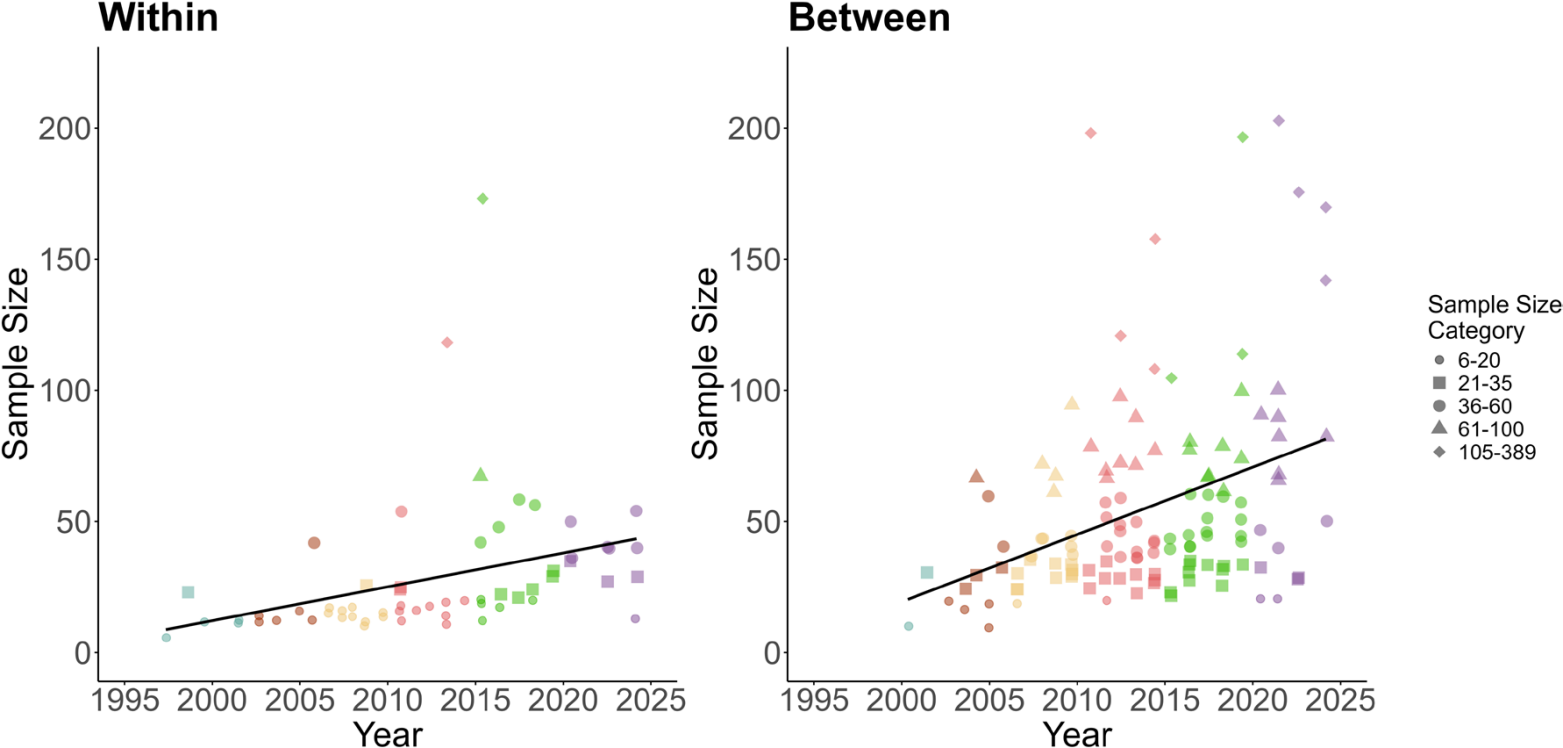
Sample Size Development. Sample Size over Time (slightly jittered), separately for within and between‐subjects designs. Color codes the (ad‐hoc) categories for year, shapes the (ad‐hoc) categories for sample size.

### 2.4 Frequency of Preregistration

Across all eligible studies (i.e., not restricted to the subsample used for the other analyses), 22 (4%) mentioned a preregistration or were conducted as registered reports. However, only 13 (2%) of those did provide a preregistered analysis plan of their EEG data (regardless of any deviations). Considering studies from 2015 onwards, only 13 (4%) out of 295 studies provided a preregistration that included the outline of (some) pre‐processing steps. Nevertheless, the proportion of studies reporting such preregistrations has steadily increased over time (2020: 3%; 2022: 10%; 2025: 15%).

### 2.5 Variance in Recording, Preprocessing & Quantification Choices

Across all steps, methodological choices varied considerably between the 202 coded studies. Notably, no two studies used an identical full preprocessing or quantification pipeline (beside two studies analyzing data from the same project). This was also true for studies with the same first or last author, indicating that substantial variance does not only occur across labs and authors. Even when reduced to a core set of preprocessing steps (i.e., referencing, filtering, bad channel handling, ocular/artifact correction), 163 unique pipelines were identified (when grouping the filter cutoff, this number decreased only slightly to 147). Likewise, for key aspects of ERN quantification (i.e., time window length/center, baseline, electrodes, method of amplitude detection), 179 unique combinations of choices were identified (grouping the baseline and ERN time windows yielded 160 unique pipelines).

Nevertheless, some methodological choices were more common across studies, as indicated by lower entropy. For example, many studies did not screen/correct for bad channels (82.18%, *H* = 0.68, Figure 3B.8) and referenced to the mastoids (51.49%, *H* = 0.68, Figure 3B.1). While almost all studies applied some correction for artifacts or blinks (98.51%, *H* = 0.11, Figure 3B.7), the exact approaches varied a lot. Across all, regression‐based algorithms to correct for eye movements and blinks (50.00%, *H* = 0.76, Figure 3B.6) and objective automated artifact identification procedures (50.50%, *H* = 0.88, Figure 3B.5) seemed most often applied. The choice showing the highest variability was the cutoff frequencies of the high‐pass filters, with 0.1 Hz as the most common one (43.56%, *H* = 0.90, Figure 3B.3).

**FIGURE 3:**
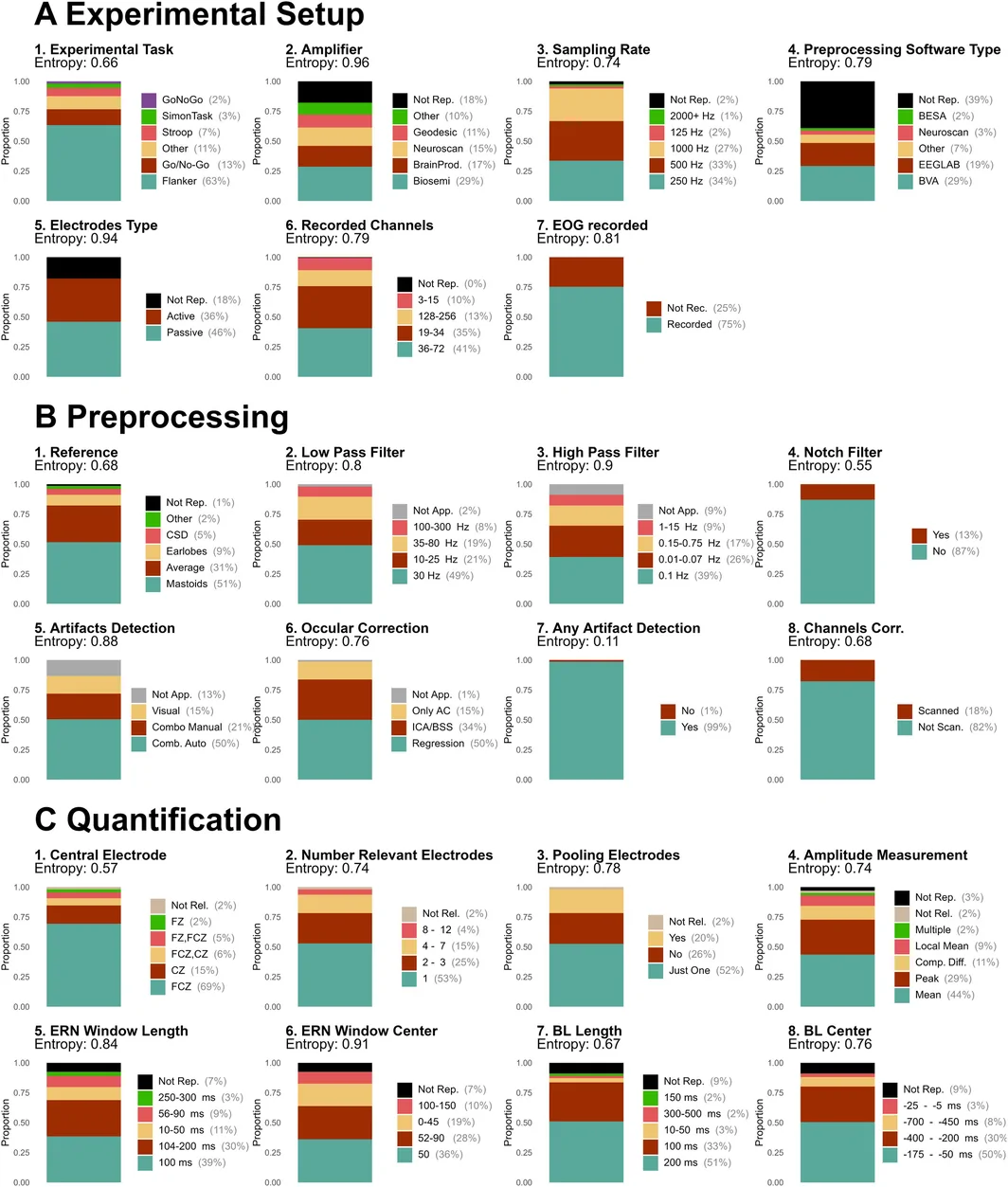
Descriptive Plot of Choices per Step. Stacked barplots together with calculated entropy illustrating the distribution of choices for (A) recording setup, (B) preprocessing, and (C) ERN quantification. Percentages indicate the proportion of studies assigned to each category. BL = baseline window. Not Rep. = Not Reported (but expected to be reported, e.g., recording setup information). Not App. = Not Applied (or optional preprocessing step not reported). Not Rel. = Not Relevant (e.g., electrode for topographical analyses).

In terms of ERN quantification, we noticed quite high entropy values over all: the lowest were found for the central electrode, where FCz was most commonly used (69.31%, *H* = 0.57, Figure 3C.1, Figure 4C), the length of the baseline, where a 200 ms window was most common (50.99%, *H* = 0.61, Figure 3C.7, Figure 4B), and how the ERN amplitude was measured, mostly as mean over the time window (43.56%, *H* = 0.74, Figure 3C.4). Highest entropy values were found regarding the time window where the ERN was quantified, often within a 100 ms long time window (38.61%, *H* = 0.84, Figure 3C.5, Figure 4A) centered around 50 ms after the response (36.14%, *H* = 0.91, Figure 3C.6). For an overview of the distribution for each relevant methodological choice, see Figure 3.

**FIGURE 4:**
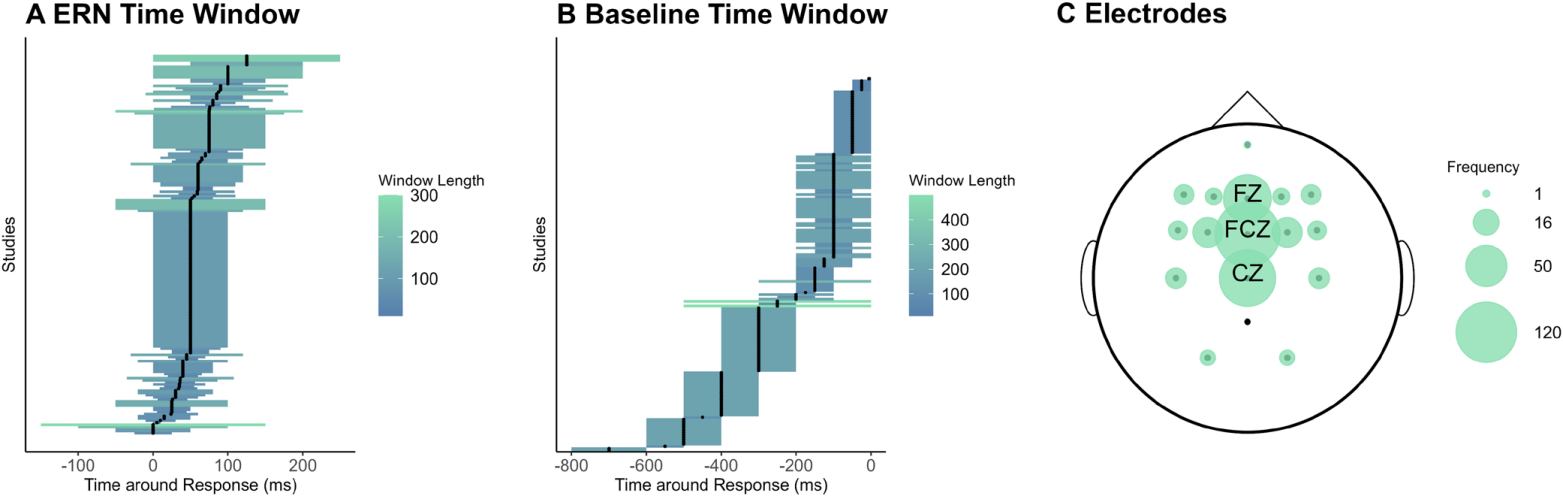
ERN Window, Baseline Window, and Electrodes Reported. (A) First panel shows the reported window to quantify the ERN component (sorted by the center of the reported window). Color encodes window length (lighter colors indicate longer windows). (B) The second panel shows the baseline in a similar way. (C) The third panel shows the reported electrodes used to quantify the ERN (i.e., excluding parietal clusters and electrode position 5–8 (if used) for comparison). Size of the circle indicates the number of studies using this particular electrode (possibly among other electrodes).

### 2.6 Explaining Variability

Below we report a selection of associations we deemed particularly interesting. Although conditioning on significant predictors led to increases in entropy for some individual decisions and decreases for others (see Table S2), the average entropy across all decisions changed only minimally (M = 0.78 without explanatory variables vs. M = 0.76), indicating that substantial methodological variability remained unaccounted for. For a detailed description of entropy values at each step, see Table S2. Other significant combinations not reported below seemed less plausible and are reported in Table S1 and illustrated in Figures S2–S17. These include, for example, associations between amplifier type and amplitude measures or baseline settings, as well as between electrode type and the correction of bad channels.

#### 2.6.1 Time Trends

As seen in Figure 5, entropy changed slightly over the years for the different choices. There was continuously high entropy for choices related to the experimental setup (*H*_AV 95–00_ = 0.83; *H*_AV 21–25_ = 0.75, Figure 5A) and the preprocessing of the data (*H*_AV 95–00_ = 0.84; *H*_AV 21–25_ = 0.76, Figure 5B). In comparison, the entropy how the ERN was quantified was very high at the beginning but seemed to decrease slightly over recent years (Time Window: *H*_AV 95–00_ = 0.91; *H*_AV 21–25_ = 0.777; Definition: *H*_AV 95–00_ = 0.92; *H*_AV 21–25_ = 0.67, Figure 5C,D).

**FIGURE 5:**
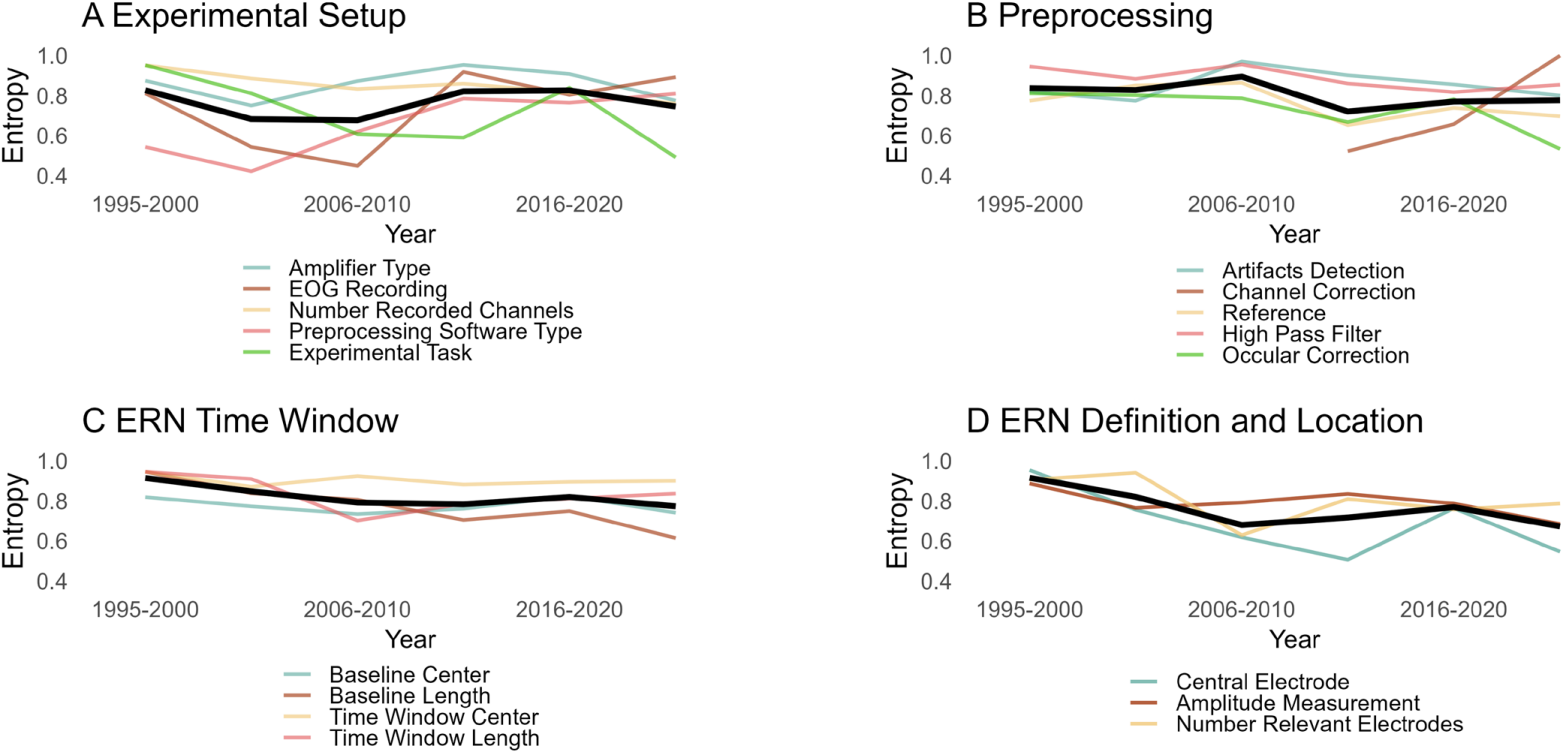
Entropy for Different Steps per Year. Entropy across years for (A) experimental setup, (B) preprocessing, (C) ERN time window, and (D) ERN definition and location. The black line represents the average of the illustrated steps.

Regarding amplifier vendors, two patterns emerged. First, amplifier details were reported only occasionally in the past (2001–2005: 37.5%), whereas they are now consistently documented (2021–2025: 100%, see Figure S3.2). Second, while there was considerable diversity among vendors in earlier years, the field has become increasingly concentrated over the past 5 years, with BioSemi (2021–2025: 50%) and Brain Products now dominating (2021–2025: 28.57%; *𝜒* ^*2*^(25) = 93.32, *p* ≤ 0.001, *R* ^*2*^ = 0.13 [0.12–0.22]; see Figure S3.2). A similar pattern was observed for the software used to analyze the data: reporting of software details was infrequent in the early 2000s (2001–2005: 12.5%), but is now almost always documented (2021–2025: 88.1%, see Figure S3.6). Between 2016 and 2020, Brain Vision Analyzer was the predominant software (49.06%), with EEGLAB becoming predominant in subsequent years (54.76%; *𝜒* ^*2*^(25) = 107.18, *p* ≤ 0.001, *R* ^*2*^ = 0.19 [0.16–0.28]).

We observed clear temporal trends in EEG preprocessing choices. Sampling rates increased over the years (*𝜒* ^*2*^(25) = 70.13, *p* ≤ 0.001, *R* ^*2*^ = 0.13 [0.10–0.21], see Figure S3.7) and active (compared to passive) electrode systems became popular only in the last 10 years (*𝜒* ^*2*^(10) = 66.78, *p* ≤ 0.001, *R* ^*2*^ = 0.16 [0.12–0.24], see Figure 6A.1). Ocular artifact correction methods shifted across the 30 years (*𝜒* ^*2*^(15) = 73.04, *p* ≤ 0.001, *R* ^*2*^ = 0.17 [0.14–0.28], see Figure 6A.2): rejection based on amplitude/spectral patterns was common before 2005 (43.75%), then correction of blinks using regression‐based approaches dominated thereafter peaking in the early 2010s (66.67%). But from 2021 onward ICA/BSS methods became the default (76.19%). Correction/identification of bad channels was not applied before 2010, but became gradually more frequent thereafter and was used in approximately half of the studies published between 2021 and 2025 (*𝜒* ^*2*^(5) = 45.88, *p* ≤ 0.001, *R* ^*2*^ = 0.24 [0.16–0.37], see Figure S3.16).

**FIGURE 6:**
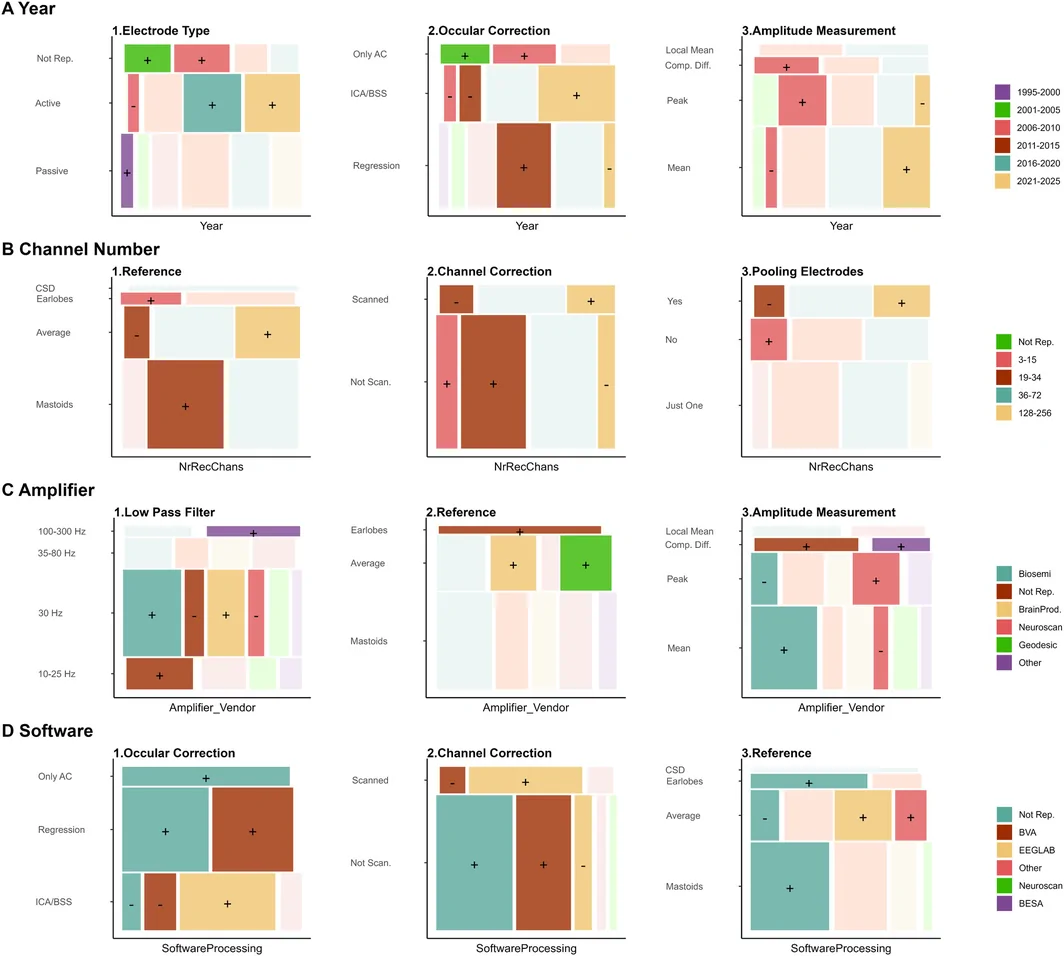
Mosaic Plots of Shared Occurrence of Multiple Choices at Different Steps. Mosaic plots illustrating the joint distribution of selected methodological choices according to (A) year, (B) channel number, (C) amplifier, and (D) preprocessing software. The height of each row reflects the marginal frequency of the corresponding y‐axis category. The width of each rectangle reflects the relative distribution of that y‐axis category across levels of the x‐axis variable. Blue indicates the most frequent category of the x‐axis variable. Bold colors and +/− symbols indicate combinations that were significantly over‐ or under‐represented (Pearson residuals of at least ±2). Only cells with at least five observations are displayed.

For ERN quantification, amplitude quantification in terms of a mean over the time window became more popular than other methods (e.g., 2021–2025: peak 11.90%, mean 59.52%; *𝜒* ^*2*^(30) = 68.94, *p* ≤ 0.001, *R* ^*2*^ = 0.12 [0.11–0.20], see Figure 6A.3). The ERN time window shifted closer to response onset (*𝜒* ^*2*^(20) = 65.10, *p* ≤ 0.001, *R* ^*2*^ = 0.11 [0.10–0.18]) and the most common length became 100 ms (2021–2025: 52.38%, *𝜒* ^*2*^(25) = 70.41, *p* ≤ 0.001, *R* ^*2*^ = 0.12 [0.10–0.20], see Figure S3.22–23, Figure S2.9–10).

#### 2.6.2 Recording Session

Beyond time trends, features of the experimental context were associated with several preprocessing choices (see Table S1). Montage size had broad effects, for example on handling of bad channels (*𝜒* ^*2*^(4) = 21.72, *p* ≤ 0.001, *R* ^*2*^ = 0.12 [0.06–0.21], see Figure 6B.2). High‐density EEG (> 72 electrodes) studies screened/repaired bad channels more often (37.04%) than lower‐density recordings (26.36%). The same high‐density studies also typically used an average reference (85.19%), (*𝜒* ^*2*^(20) = 78.23, *p* ≤ 0.001, *R* ^*2*^ = 0.16 [0.13–0.25], see Figure 6B.1). Although very small montages (< 16 electrodes) relied on average reference less often, a small fraction still did (4.76%).

Amplifier vendors also showed a few associations. Low‐pass cut‐offs clustered around 30 Hz for Brain Products and BioSemi systems (63.44%), whereas other systems exhibited wider variation (*𝜒* ^*2*^(20) = 54.02, *p* ≤ 0.001, R^2^ = 0.10 [0.09–0.20], see Figure 6C.1). Reference choices also differed between amplifier vendors (*𝜒* ^*2*^(25) = 75.36, *p* ≤ 0.001, *R* ^*2*^ = 0.15 [0.13–0.25], see Figure 6C.2): Brain Products and Geodesic/EGI systems had a stronger tendency to use an average reference (81.82%), while others did not show such a convergence.

The preprocessing software environment was related to ocular‐artifact handling (*𝜒* ^*2*^(15) = 132.62, *p* ≤ 0.001, *R* ^*2*^ = 0.31 [0.25–0.42], see Figure 6D.1). Regression‐based correction was most common overall (50.00%) and particularly prevalent in BrainVision Analyzer pipelines (76.27%), whereas EEGLAB pipelines predominantly relied on source‐separation approaches (e.g., ICA, 92.31%).

Lastly, sample size had little effect on the analyses, except that larger samples were associated with a higher likelihood of bad‐channel correction (*𝜒* ^*2*^(4) = 19.65, *p* = 0.001, *R* ^*2*^ = 0.10 [0.04–0.23], see Figure S9.8).

#### 2.6.3 Differences in the Order of Preprocessing Steps

Preprocessing order varied substantially across the 202 included studies. In total, 86 unique preprocessing‐step orderings were identified. Most pipeline orders were used by only a small number of studies, with frequencies ranging from 1 to 14 studies per pipeline (*M* = 2.35, SD = 2.48, see Figure 7A for the most frequent orders). The mean pairwise Levenshtein distance between order strings was 2.88 (SD = 1.03), indicating that approximately three edit operations were required, on average, to transform one pipeline sequence into another. This is a large degree of divergence given the five‐step pipeline examined. The transition matrix (Figure 7B) revealed modest local regularities despite the high global diversity of preprocessing pipelines. Referencing, filtering, and epoching were all common entry points into the preprocessing workflow, with no single step serving as a universal starting operation (24%–33%). Nevertheless, filtering was frequently followed by epoching (30%), and epoching was often followed by artifact correction procedures (36%), suggesting partial agreement regarding the placement of artifact‐related processing later in the pipeline. Transition probabilities were generally diffuse, however, with no transition exceeding a probability of 36%, further underscoring the absence of a dominant preprocessing sequence across studies. Normalized Shannon entropy of the order‐string distribution was 0.919, confirming that no canonical ordering dominated the literature and that the observed orders were distributed nearly uniformly across all attested variants.

**FIGURE 7:**
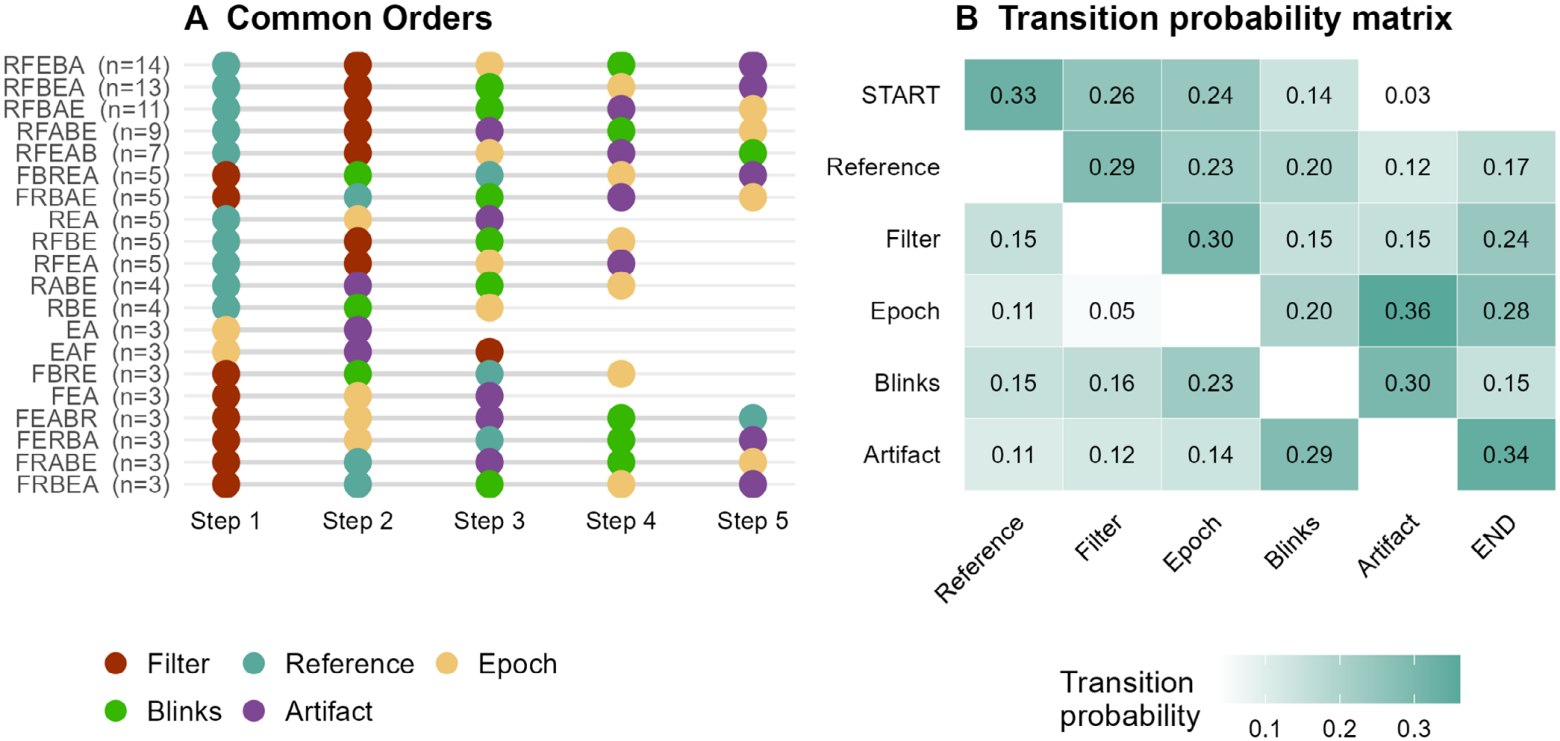
Diversity and Transition Structure of Preprocessing Pipelines across Studies. (A) The 20 most common preprocessing orders identified across studies. Colored circles indicate preprocessing steps, connected to illustrate the sequential order of operations within each pipeline. Labels indicate the preprocessing sequence and the number of studies using that order (abbreviated by first letter). (B) Transition probability matrix summarizing the likelihood of transitioning from one preprocessing step to the next. Rows represent the current preprocessing step and columns represent the subsequent step. Cell values indicate transition probabilities, with darker shading reflecting higher agreement across studies. “START” denotes the initial preprocessing step and “END” denotes the final preprocessing step in a pipeline.

## 3 Discussion

The present systematic review shows that methodological heterogeneity in the preprocessing and analysis of the ERN (Error Related Negativity) is extremely high, with no two identical analysis routines across 202 papers. A few time‐trends emerged, and several features of the recording setup explained some of the variability. Nevertheless, substantial methodological heterogeneity remained unexplained after these factors were considered. Finally, whereas slight increases in sample sizes did emerge across three decades, statistical power still remains unsatisfactory for many of the recent studies (especially for between‐subjects designs) and detailed preregistrations are still extremely rare and just becoming more frequent in the last year. The following sections discuss these findings in more detail.

### 3.1 Heterogeneity in ERN Preprocessing and Analysis Pipelines

Matching prior observations for N400 and P300 components (i.e., stimulus‐locked ERPs; Jacobsen et al. 2025; Šoškić et al. 2022), we found considerable variability in preprocessing and analysis pipelines for the ERN (i.e., a response‐locked ERP) across studies. In fact, no two studies shared the exact same pipeline and variability was observed even among studies sharing the same first or last author. This suggests that methodological heterogeneity is not restricted to specific ERP components or settings but instead may be a more general feature of ERP research. For the ERN in particular, prior work suggests that some commonly used analytic approaches often yield broadly similar psychometric properties and associations (e.g., with anxiety), although systematic differences between specific analytic choices remain (Klawohn et al. 2020). This highlights that not all sources of variability necessarily translate into meaningful differences in inference. However, methodological choices can influence the resulting ERP estimates (e.g., Beauducel et al. 2024; Paul et al. 2022; Šoškić et al. 2024), which ultimately complicates between‐study comparisons, meta‐analyses, and cumulative science. In this context, systematically evaluating the robustness of findings across reasonable analytic choices within a single dataset (e.g., alternative time windows, electrode selection, or referencing approaches) represents a promising strategy to clarify when analytic flexibility meaningfully affects conclusions. The combination of limited methodological consensus and extensive analytical flexibility places early‐career researchers in a particularly vulnerable position, requiring them to commit to influential analytical choices without clear guidance (Garrett‐Ruffin et al. 2021). Systematic comparisons of more common preprocessing/analysis options with respect to reliability and validity of the ERP measures are needed to clarify which approaches are appropriate for different tasks, recording conditions, populations, data‐quality profiles, and research questions. Such work could support clearer recommendations and decision rules while recognizing that more than one pipeline may remain defensible.

Beyond variability in the selection and parameterization of preprocessing steps, substantial heterogeneity was also observed in the ordering of preprocessing operations. Although only five common preprocessing steps were considered, studies already exhibited a large number of distinct pipeline sequences and considerable variability in transition structure. While some preprocessing orders appeared more common than others (e.g., starting with referencing, filtering before epoching etc.), most pipelines remained relatively unique. This suggests that preprocessing order itself represents an additional and often underappreciated source of analytic flexibility in ERP research that may further complicate reproducibility and between‐study comparability.

### 3.2 Evolution of Pipeline Choices Across Three Decades (1995–2025)

Several observed trends align with improvements in amplifiers/recording systems, new benchmarks for preprocessing software, and of course data storage, which collectively enabled higher sampling rates and denser montages. Availability might explain findings such as that active electrodes (as opposed to passive systems) are becoming more frequent since 2015 (after Biosemi introduced the first commercial EEG system with active electrodes in 2002, followed by brain products 2005). Since the 1990s, ocular correction was most dominantly performed with regression‐based techniques that use frontal EOG (electrooculography) to model and subtract ocular activity from each EEG channel. These techniques are favored for their simplicity, transparency, and wide dissemination (e.g., through Gratton et al. 1983). In parallel and in particular during the last 5 years, more analytically complex approaches became more popular, possibly due to better computational efficiency. These techniques aim to separate EEG data into statistically independent components/sources, to identify and remove ocular (or not‐brain related) components. Not only do these approaches need more recorded data (in particular with respect to sampling rate, see Frank et al. 2025), but are now also facilitated by mainstream toolboxes to guide component selection, e.g., EEGLAB plug‐ins such as ICLabel (Pion‐Tonachini et al. 2019) or SASICA (Chaumon et al. 2015) or BESA's correction based on Berg and Scherg (1994).

Time trends were also observed for the quantification of the ERN. Researchers increasingly prefer mean over peak measures, possibly driven by guidance emphasizing better signal‐to‐noise ratio and reliability of mean amplitudes (Clayson et al. 2013; Luck 2014). However, prior work suggests that these scoring approaches can yield comparable internal consistency in some contexts (e.g., Olvet and Hajcak 2009; Sandre et al. 2020), suggesting that this shift is not solely attributable to reliability considerations. Instead, it may also reflect practical advantages of mean amplitude measures, including their straightforward implementation in automated analysis pipelines and reduced dependence on experimenter judgment. Interestingly, researchers also chose a time window closer to the response onset while shifting the baseline to an earlier time point, which aligns with current perspectives on the generation of the ERN component (Gehring et al. 2018).

Note, however, that all observed time trends were relatively weak, and the entropy of methodological choices remained high even in the most recent years. This stands in contrast to the reduced variability observed for certain aspects of the recording setup (e.g., amplifier vendors and preprocessing software), suggesting that convergence at the level of hardware and software has not resulted in broader methodological standardization. Consequently, there is little evidence at methodological practices have converged over time, despite repeated calls for clearer methodological guidance. (Keil et al. 2014; Picton et al. 2000). This state of affairs is likely due to a lack of studies systematically comparing the consequences of preprocessing/analysis choices for the reliability and validity of ERPs of interest (but see Clayson 2024; Klawohn et al. 2020). For many methodological decisions it is currently still unclear, whether some alternatives are superior to others or whether their suitability depends on features of the task, montage, population, artifact profile, error count, or research question. However, the fact that it is somewhat common to not even report all the methodological details recommended (Keil et al. 2014), suggests that ERP researchers still tend to underestimate the potential impact of methodological variability. Recent initiatives such as ARTEM‐IS, which promote comprehensive and standardized reporting, therefore represent an important step toward greater transparency and reproducibility (Šoškić et al. 2025). Greater convergence in transparent reporting and clearer guidance on relevant decision rules may be more attainable and informative than convergence on one universally applicable pipeline.

### 3.3 Influence of Experimental Task and Recording/Laboratory Setup on Variability

Features of the recording setup explained some variability in preprocessing pipelines, but their effects were modest. More traceable associations were found for the number of recorded channels and choices how to reference and clean the data: More recorded channels were linked to a higher probability of using an average reference, consistent with guidance to avoid average reference in sparse montages (Junghöfer et al. 1999; Nunez and Srinivasan 2006). With more channels, studies screened/interpolated bad channels more routinely, unsurprising given that the chance of at least one faulty electrode rises with montage size. Larger sample sizes also coincided with more frequent screening for bad channels, consistent with the growing use of automated/semi‐automated quality controls to scale pipelines.

By contrast, amplifier vendors, electrode types (active vs. passive), and analysis software explained relatively few aspects of the preprocessing pipeline. For instance, commonly used amplifier systems (e.g., Brain Products, BioSemi) tended to converge on around 30 Hz low‐pass cutoffs, consistent with community conventions (Zhang et al. 2024) and vendor templates. Less frequently used systems showed greater heterogeneity, likely reflecting diverse standard operating procedures across labs. On the other hand, artifact handling was more influenced by the analysis software used: BrainVision Analyzer pipelines more often reported regression‐based correction for ocular artifacts, whereas EEGLAB pipelines more often used ICA/BSS, consistent with EEGLAB's long‐standing emphasis on ICA workflows and implemented tools (e.g., ICLabel. Pion‐Tonachini et al. 2019).

Overall, vendor defaults, analysis software, and especially montage size nudge researchers toward particular choices but do not strongly determine them. More importantly, many choices were not well‐explained by features of either experimental task or the recording/laboratory setup (e.g., how non‐ocular artifacts were handled, high‐pass filter cutoffs, or where and when the ERN was quantified). Where we found associations, the entropy remained high even after controlling for relevant variables. Although some variability may reflect data quality, task characteristics or unmeasured setup features, a substantial proportion of methodological variability remained unexplained by the factors examined in the present study.

### 3.4 Improvements of Sample Size and the Percentage of Preregistered Studies

We observed a steady increase in sample size over time, an encouraging trend for statistical power and estimation precision (cf. Clayson et al. 2019). Note, however, that even in the most recent years the median sample size of 56 for within‐subject effects and 115 for between‐subjects effects (total number) only provides sufficient power of 0.80 to detect relatively large effects. For within‐subject designs this means mean difference of *d* = 0.4, and for between‐subjects analysis a correlation of *r* = 0.3. In particular the latter is problematic, as the typical effect size within individual difference research tends to be closer to *r* = 0.2 (Gignac and Szodorai 2016). This suggests that insufficient power in a significant percentage of studies remains a problem even in recent years. Furthermore, preregistration that specifies each preprocessing and analysis step remains rare in this literature, mirroring the state of affairs in the wider field of Psychology (Hardwicke et al. 2022, 2024; Wacker et al. 2026). Thus, ERN researchers often retain considerable analytical flexibility, which may influence the outcomes of EEG analyses (e.g., Beauducel et al. 2024; Clayson 2024; Klawohn et al. 2020; Paul et al. 2022).

### 3.5 Reporting Fidelity

Reporting fidelity remains a bottleneck in ERN research. As in the N400 literature, audits have documented inconsistent and incomplete method reporting despite widely disseminated EEG/MEG guidance (e.g., Keil et al. 2014). Even within our limited variable set, hardware/software provenance and the specifics of artifact‐correction (extent of manual vs. automated steps) were often underspecified. Moving beyond narrative checklists, adopting standardized, tool‐supported formats such as ARTEM‐IS for ERP (Šoškić et al. 2025), is needed to improve transparency, comparability, and meta‐analytic reuse.

### 3.6 Limitations

The current study features the first systematic review of ERN preprocessing and analysis methods employed across three decades. The analyses were based on a year‐stratified subsample of eligible studies, specifically studies involving adults performing conventional stimulus–response tasks. Consequently, the resulting estimates may not fully represent either all studies within the defined sampling frame or the broader literature. Furthermore, inter‐rater agreement for the information extracted from each study was high, though not perfect, thereby introducing an additional, albeit minor, source of unsystematic variance. It should also be noted that some of the observed heterogeneity may reflect within‐task parameters (e.g., trial counts, error rates), features of the EEG data (noise, number of epochs) or other features of the recording/laboratory setup not captured in the present study. Examining these factors represents an important direction for future work. Another limitation relates to the definition of categories, in particular for continuous variables. Because entropy estimates depend on category definitions, the observed diversity values are necessarily influenced by binning decisions for continuous methodological variables. Bins were chosen based on observed distributions and methodological interpretability. However, exploratory analyses using quantile‐based categorizations produced qualitatively similar patterns of relative diversity across preprocessing and analysis steps (see Table S6). Moreover, because categories such as “Not reported” and “Not applied” were retained in the analyses, entropy comparisons across variables should be interpreted cautiously, as diversity estimates are also influenced by reporting completeness and category granularity. Moreover, given the broad scope of the analyses and the large number of evaluated associations, the present findings should primarily be interpreted as exploratory and descriptive in nature. Accordingly, individual associations should be interpreted cautiously until replicated in future work. Finally, variability would be less indicative of excessive analytic flexibility if it primarily occurred between laboratories that consistently adhere to fixed processing pipelines. However, the present sampling strategy was not designed to systematically capture multiple studies from the same laboratory, limiting our ability to directly assess within‐laboratory consistency. Although we observed some methodological differences among studies conducted by the same authors, this evidence is necessarily limited, and future work specifically targeting within‐laboratory practices would be needed.

## 4 Conclusions

This systematic review of a year‐stratified sample of 202 eligible studies published between 1995 and 2025 revealed significant variability in preprocessing and analysis pipelines of the error‐related negativity matching prior observations for stimulus‐locked ERPs (Jacobsen et al. 2025; Šoškić et al. 2024). Some systematic time trends, and several associations between preprocessing/analysis decisions and features of the recording setup were found. Nevertheless, most of the methodological variability remains unaccounted for. Because some methodological choices can affect the resulting estimates (e.g., Beauducel et al. 2024; Paul et al. 2022; Šoškić et al. 2024) methodological variability across studies can complicate between‐study comparisons, meta‐analyses, and cumulative science. This situation may be particularly challenging for early‐career researchers, who must make methodological decisions in a field where consensus remains limited. Despite the availability of detailed recommendations (Paul et al. 2021; Binney et al. 2025), sufficiently specific preregistrations were extremely rare in this literature (see also Wacker et al. 2026). Furthermore, although sample sizes increased over time, statistical power often remained limited for the relatively small effects typically expected in inter‐individual research. Taken together, the sampled literature on adult ERN studies using response‐conflict tasks and ERP analyses remains methodologically diverse, with limited convergence in analytic practice, rare use of detailed preregistration, and continuing limitations in statistical power. Clearer documentation of analytic decisions, systematic evaluation of results across plausible pipelines, and further development of methodological guidance on when different pipelines are appropriate would facilitate the interpretation, comparison, and synthesis of ERN findings across studies.

## Author Contributions

**Jan Wacker:** conceptualization, methodology, validation, writing – review and editing, funding acquisition. **Katharina Paul:** conceptualization, methodology, formal analysis, investigation, data curation, visualization, writing – original draft, writing – review and editing. **Johanna Paping:** investigation, data curation, writing – review and editing.

## Funding

The study was supported by the German Research Foundation (WA 2593, PA 4014/2–2).

## Conflicts of Interest

The authors declare no conflicts of interest.

## Supporting information


**Table S1:** Overview X^2^ Tests.
**Table S2:** Remaining Entropy after Controlling for several Variables.
**Table S3:** Inter Rater Reliability.
**Table S4:** List of all coded Variables.
**Figure S1:** Distribution of how continuous variables were grouped.
**Figure S2:** Mosaic Plots of shared occurrence of multiple choices.
Distribution of choices for different combinations presented as a mosaic plot. The color blue indicates the most frequent category of the *x* axis (across all levels of the *y* axis). Similarly, the row's height on the *y* axis indicates the frequency of the categories on the *y* axis (across all levels of the *x* axis). The width of each rectangle shows the relative distribution of one category on the *y* axis across the levels of the *y* axis. Bold colors and +/− indicate combinations significantly over‐ or under‐represented (as defined by a $X^{2}$ test). Only cells with at least 5 occurrences are shown. Plots surrounded by a frame were significant across our criteria (*p* < 0.001, *V/R* ^*2*^ > 0.1).
**Figure S3:** Year.
**Figure S4:** Low pass filter.
**Figure S5:** High pass filter.
**Figure S6:** Reference.
**Figure S7:** Channel correction.
**Figure S8:** Occular correction.
**Figure S9:** Artifacts detection.
**Figure S10:** Central electrode.
**Figure S11:** Number relevant electrodes.
**Figure S12:** Pooling electrodes.
**Figure S13:** Baseline center.
**Figure S14:** Baseline length.
**Figure S15:** Time window center.
**Figure S16:** Time window length.
**Figure S17:** Amplitude measurement.

## Data Availability

The data that support the findings of this study are openly available in OSF at https://osf.io/ak46v/.

## Endnotes

^1^
The *R* ^2^ values are not comparable between logistic/multinomial and linear models and were used only as a pragmatic selection criterion, not as indicating comparable effect sizes across model types.

## References

[psyp70402-bib-0001] Beauducel, A. , V. Scheuble‐Cabrera , J. Hennig , et al. 2024. “The Association of Dispositional Anxiety With the no‐Go N2 Under Relaxation Instruction vs. Speed/Accuracy Instruction.” Biological Psychology 192: 108850. 10.1016/j.biopsycho.2024.108850 39074541

[psyp70402-bib-0002] Berg, P. , and M. Scherg . 1994. “A Multiple Source Approach to the Correction of Eye Artifacts.” Electroencephalography and Clinical Neurophysiology 90, no. 3: 229–241. 10.1016/0013-4694(94)90094-9 7511504

[psyp70402-bib-0003] Binney, R. J. , L. J. Smith , S. Rossit , et al. 2025. “Practical Routes to Preregistration: A Guide to Enhanced Transparency and Rigour in Neuropsychological Research.” Brain Communications 7, no. 3: fcaf162. 10.1093/braincomms/fcaf162 PMC12066951 40357015

[psyp70402-bib-0004] Chaumon, M. , D. V. Bishop , and N. A. Busch . 2015. “A Practical Guide to the Selection of Independent Components of the Electroencephalogram for Artifact Correction.” Journal of Neuroscience Methods 250: 47–63. 10.1016/j.jneumeth.2015.02.025 25791012

[psyp70402-bib-0005] Clayson, P. E. 2024. “Beyond Single Paradigms, Pipelines, and Outcomes: Embracing Multiverse Analyses in Psychophysiology.” International Journal of Psychophysiology 197: 112311. 10.1016/j.ijpsycho.2024.112311 38296000

[psyp70402-bib-0006] Clayson, P. E. , S. A. Baldwin , and M. J. Larson . 2013. “How Does Noise Affect Amplitude and Latency Measurement of Event‐Related Potentials (ERPs)? A Methodological Critique and Simulation Study.” Psychophysiology 50, no. 2: 174–186. 10.1111/psyp.12001 23216521

[psyp70402-bib-0007] Clayson, P. E. , S. A. Baldwin , H. A. Rocha , and M. J. Larson . 2021. “The Data‐Processing Multiverse of Event‐Related Potentials (ERPs): A Roadmap for the Optimization and Standardization of ERP Processing and Reduction Pipelines.” NeuroImage 245: 118712. 10.1016/j.neuroimage.2021.118712 34800661

[psyp70402-bib-0008] Clayson, P. E. , K. A. Carbine , S. A. Baldwin , and M. J. Larson . 2019. “Methodological Reporting Behavior, Sample Sizes, and Statistical Power in Studies of Event‐Related Potentials: Barriers to Reproducibility and Replicability.” Psychophysiology 56, no. 11: e13437. 10.1111/psyp.13437 31322285

[psyp70402-bib-0009] Clayson, P. E. , K. A. Carbine , J. L. Shuford , J. B. McDonald , and M. J. Larson . 2025. “A Registered Report of Preregistration Practices in Studies of Electroencephalogram (EEG) and Event‐Related Potentials (ERPs): A First Look at Accessibility, Adherence, Transparency, and Selection Bias.” Cortex 185: 253–269. 10.1016/j.cortex.2025.02.008 40107168

[psyp70402-bib-0010] Clayson, P. E. , J. B. Mcdonald , B. Park , et al. 2025. “Registered Replication Report of the Construct Validity of the Error‐Related Negativity (ERN): A Multi‐Site Study of Task‐Specific ERN Correlations With Internalizing and Externalizing Symptoms.” Psychophysiology 62, no. 1: e14496. 10.1111/psyp.14496 38155370

[psyp70402-bib-0011] Del Giudice, M. , and S. W. Gangestad . 2021. “A Traveler's Guide to the Multiverse: Promises, Pitfalls, and a Framework for the Evaluation of Analytic Decisions.” Advances in Methods and Practices in Psychological Science 4, no. 1: 2515245920954925.

[psyp70402-bib-0012] Frank, G. , S. Y. Shirazi , J. Palmer , G. Cauwenberghs , S. Makeig , and A. Delorme . 2025. “Quantifying Data Requirements for EEG Independent Component Analysis Using AMICA.” arXiv Preprint arXiv 2506: 10156.

[psyp70402-bib-0013] Garrett‐Ruffin, S. , A. C. Hindash , A. N. Kaczkurkin , et al. 2021. “Open Science in Psychophysiology: An Overview of Challenges and Emerging Solutions.” International Journal of Psychophysiology 162: 69–78. 10.1016/j.ijpsycho.2021.02.005 33556468

[psyp70402-bib-0014] Gehring, W. J. , M. G. H. Coles , D. E. Meyer , and E. Donchin . 1990. “The Error‐Related Negativity: An Event‐Related Brain Potential Accompanying Errors.” Psychophysiology 27: S34.

[psyp70402-bib-0015] Gehring, W. J. , B. Goss , M. G. Coles , D. E. Meyer , and E. Donchin . 2018. “The Error‐Related Negativity.” Perspectives on Psychological Science 13, no. 2: 200–204. 10.1177/1745691617715310 29592655

[psyp70402-bib-0016] Gignac, G. E. , and E. T. Szodorai . 2016. “Effect Size Guidelines for Individual Differences Researchers.” Personality and Individual Differences 102: 74–78.

[psyp70402-bib-0017] Gratton, G. , M. G. Coles , and E. Donchin . 1983. “A New Method for Off‐Line Removal of Ocular Artifact.” Electroencephalography and Clinical Neurophysiology 55, no. 4: 468–484. 10.1016/0013-4694(83)90135-9 6187540

[psyp70402-bib-0018] Hardwicke, T. E. , R. T. Thibault , B. Clarke , et al. 2024. “Prevalence of Transparent Research Practices in Psychology: A Cross‐Sectional Study of Empirical Articles Published in 2022.” Advances in Methods and Practices in Psychological Science 7, no. 4: 25152459241283477.

[psyp70402-bib-0019] Hardwicke, T. E. , R. T. Thibault , J. E. Kosie , J. D. Wallach , M. C. Kidwell , and J. P. A. Ioannidis . 2022. “Estimating the Prevalence of Transparency and Reproducibility‐Related Research Practices in Psychology (2014–2017).” Perspectives on Psychological Science 17, no. 1: 239–251. 10.1177/1745691620979806 PMC8785283 33682488

[psyp70402-bib-0020] Hohnsbein, J. , M. Falkenstein , and J. Hoormann . 1989. “Error Processing in Visual and Auditory Choice Reaction Tasks.” Journal of Psychophysiology 3: 320.

[psyp70402-bib-0021] Jacobsen, N. S. , D. Kristanto , S. Welp , Y. C. Inceler , and S. Debener . 2025. “Preprocessing Choices for P3 Analyses With Mobile EEG: A Systematic Literature Review and Interactive Exploration.” Psychophysiology 62, no. 1: e14743. 10.1111/psyp.14743 PMC11656290 39697161

[psyp70402-bib-0022] Junghöfer, M. , T. Elbert , D. M. Tucker , and C. Braun . 1999. “The Polar Average Reference Effect: A Bias in Estimating the Head Surface Integral in EEG Recording.” Clinical Neurophysiology 110, no. 6: 1149–1155. 10.1016/s1388-2457(99)00044-9 10402104

[psyp70402-bib-0023] Kappenman, E. S. , and S. J. Luck . 2016. “Best Practices for Event‐Related Potential Research in Clinical Populations.” Biological Psychiatry: Cognitive Neuroscience and Neuroimaging 1, no. 2: 110–115. 10.1016/j.bpsc.2015.11.007 PMC4797328 27004261

[psyp70402-bib-0024] Keil, A. , S. Debener , G. Gratton , et al. 2014. “Committee Report: Publication Guidelines and Recommendations for Studies Using Electroencephalography and Magnetoencephalography.” Psychophysiology 51, no. 1: 1–21. 10.1111/psyp.12147 24147581

[psyp70402-bib-0025] Klawohn, J. , A. Meyer , A. Weinberg , and G. Hajcak . 2020. “Methodological Choices in Event‐Related Potential (ERP) Research and Their Impact on Internal Consistency Reliability and Individual Differences: An Examination of the Error‐Related Negativity (ERN) and Anxiety.” Journal of Abnormal Psychology 129, no. 1: 29–37. 10.1037/abn0000458 PMC6931902 31868385

[psyp70402-bib-0026] Larson, M. J. , and K. A. Carbine . 2017. “Sample Size Calculations in Human Electrophysiology (EEG and ERP) Studies: A Systematic Review and Recommendations for Increased Rigor.” International Journal of Psychophysiology 111: 33–41. 10.1016/j.ijpsycho.2016.06.015 27373837

[psyp70402-bib-0049] Luck, S. J. 2014. An Introduction to the Event‐Related Potential Technique. MIT Press.

[psyp70402-bib-0027] Luck, S. J. , and N. Gaspelin . 2017. “How to Get Statistically Significant Effects in Any ERP Experiment (And Why You Shouldn't).” Psychophysiology 54, no. 1: 146–157. 10.1111/psyp.12639 PMC5178877 28000253

[psyp70402-bib-0028] MacCoun, R. , and S. Perlmutter . 2015. “Blind Analysis: Hide Results to Seek the Truth.” Nature 526, no. 7572: 187–189. 10.1038/526187a 26450040

[psyp70402-bib-0029] Nunez, P. L. , and R. Srinivasan . 2006. Electric Fields of the Brain: The Neurophysics of EEG. Oxford university press.

[psyp70402-bib-0030] Olvet, D. M. , and G. Hajcak . 2009. “Reliability of Error‐Related Brain Activity.” Brain Research 1284: 89–99. 10.1016/j.brainres.2009.05.079 19501071

[psyp70402-bib-0031] Page, M. J. , J. E. McKenzie , P. M. Bossuyt , et al. 2021. “The PRISMA 2020 Statement: An Updated Guideline for Reporting Systematic Reviews.” BMJ 372: n71. 10.1136/bmj.n71 PMC8005924 33782057

[psyp70402-bib-0032] Paul, K. , A. Beauducel , J. Hennig , et al. 2025. “Frontal Alpha Asymmetry as a Marker of Approach Motivation? Insights From a Cooperative Forking Path Analysis.” Journal of Personality and Social Psychology 128, no. 1: 196–210. 10.1037/pspp0000503 39570685

[psyp70402-bib-0033] Paul, K. , C. A. Short , A. Beauducel , et al. 2022. “The Methodology and Dataset of the Coscience Eeg‐Personality Project–a Large‐Scale, Multi‐Laboratory Project Grounded in Cooperative Forking Paths Analysis.” Personality Science 3, no. 1: e7177.

[psyp70402-bib-0034] Paul, M. , G. H. Govaart , and A. Schettino . 2021. “Making ERP Research More Transparent: Guidelines for Preregistration.” International Journal of Psychophysiology 164: 52–63. 10.1016/j.ijpsycho.2021.02.016 33676957

[psyp70402-bib-0035] Pavlov, Y. G. , N. Adamian , S. Appelhoff , et al. 2021. “EEGManyLabs: Investigating the Replicability of Influential EEG Experiments.” Cortex 144: 213–229. 10.1016/j.cortex.2021.03.013 33965167

[psyp70402-bib-0036] Picton, T. W. , S. Bentin , P. Berg , et al. 2000. “Guidelines for Using Human Event‐Related Potentials to Study Cognition: Recording Standards and Publication Criteria.” Psychophysiology 37, no. 2: 127–152. 10731765

[psyp70402-bib-0037] Pion‐Tonachini, L. , K. Kreutz‐Delgado , and S. Makeig . 2019. “ICLabel: An Automated Electroencephalographic Independent Component Classifier, Dataset, and Website.” NeuroImage 198: 181–197. 10.1016/j.neuroimage.2019.05.026 PMC6592775 31103785

[psyp70402-bib-0038] Sandre, A. , I. Banica , A. Riesel , J. Flake , J. Klawohn , and A. Weinberg . 2020. “Comparing the Effects of Different Methodological Decisions on the Error‐Related Negativity and Its Association With Behaviour and Gender.” International Journal of Psychophysiology 156: 18–39. 10.1016/j.ijpsycho.2020.06.016 32653551

[psyp70402-bib-0039] Saunders, B. , and M. Inzlicht . 2020. “Assessing and Adjusting for Publication Bias in the Relationship Between Anxiety and the Error‐Related Negativity.” International Journal of Psychophysiology 155: 87–98. 10.1016/j.ijpsycho.2020.05.008 32479773

[psyp70402-bib-0040] Simmons, J. P. , L. D. Nelson , and U. Simonsohn . 2011. “False‐Positive Psychology: Undisclosed Flexibility in Data Collection and Analysis Allows Presenting Anything as Significant.” Psychological Science 22, no. 11: 1359–1366. 10.1177/0956797611417632 22006061

[psyp70402-bib-0041] Šoškić, A. , V. Jovanović , S. J. Styles , E. S. Kappenman , and V. Ković . 2022. “How to Do Better N400 Studies: Reproducibility, Consistency and Adherence to Research Standards in the Existing Literature.” Neuropsychology Review 32, no. 3: 577–600. 10.1007/s11065-021-09513-4 PMC9381463 34374003

[psyp70402-bib-0042] Šoškić, A. , V. Ković , J. Algermissen , et al. 2025. “ARTEM‐IS for ERP: Agreed Reporting Template for EEG Methodology—International Standard for Event‐Related Potential Experiments.” Psychophysiology 62, no. 12: e70187. 10.1111/psyp.70187 PMC12683983 41358599

[psyp70402-bib-0043] Šoškić, A. , S. J. Styles , E. S. Kappenman , and V. Ković . 2024. “Garden of Forking Paths in ERP Research–Effects of Varying Pre‐Processing and Analysis Steps in an N400 Experiment.” Psychophysiology 61, no. 10: e14628. 10.1111/psyp.14628 38961523

[psyp70402-bib-0044] Steegen, S. , F. Tuerlinckx , A. Gelman , and W. Vanpaemel . 2016. “Increasing Transparency Through a Multiverse Analysis.” Perspectives on Psychological Science 11, no. 5: 702–712. 10.1177/1745691616658637 27694465

[psyp70402-bib-0045] Trübutschek, D. , Y. F. Yang , C. Gianelli , et al. 2024. “EEGManyPipelines: A Large‐Scale, Grassroots Multi‐Analyst Study of Electroencephalography Analysis Practices in the Wild.” Journal of Cognitive Neuroscience 36, no. 2: 217–224. 10.1162/jocn_a_02087 38010291

[psyp70402-bib-0046] Wacker, J. 2017. “Increasing the Reproducibility of Science Through Close Cooperation and Forking Path Analysis.” Frontiers in Psychology 8: 1332. 10.3389/fpsyg.2017.01332. PMC5541020 28824510

[psyp70402-bib-0047] Wacker, J. , K. Paul , and A. Scharfenecker . 2026. “Brain Association Studies in 2024: A Systematic Review of Sample Sizes and Preregistrations.” Psychophysiology 63, no. 7: e70365. 10.1111/psyp.70365 PMC13401052 42499226

[psyp70402-bib-0048] Zhang, G. , D. R. Garrett , and S. J. Luck . 2024. “Optimal Filters for ERP Research II: Recommended Settings for Seven Common ERP Components.” Psychophysiology 61, no. 6: e14530. 10.1111/psyp.14530 PMC11096077 38282093

