# Supplementary material for: Variability in Preprocessing and Analysis of the Error‐Related Negativity in Adult Response‐Conflict Studies: A Systematic Review
Source: Psychophysiology. 2026 Oct 8;63(10):e70402. doi: 10.1111/psyp.70402 (PMC13650170; doi:10.1111/psyp.70402)
Supplement: Supplementary file 1 [file PSYP-63-e70402-s001.docx]

**Supplement**

**This supplement contains:**

[Supplementary Table 1: Overview X² Tests	2](#_Toc218514430)

[Supplementary Table 2: Remaining Entropy after Controlling for several Variables	5](#_Toc218514431)

[Supplementary Table 3: Inter Rater Reliability.	6](#_Toc218514432)

[Supplementary Table 4. List of all coded Variables	7](#_Toc218514433)

[Supplementary Figure 1: Distribution of how continuous variables were grouped	9](#_Toc218514434)

[Supplementary Figure 2+: Mosaic Plots of shared occurrence of multiple choices.	10](#_Toc218514435)

*Time Trends	13-14*

*Filters	15*

*Reference	16*

*Correction for Bad Channels	16*

*Artifact Correction & Blinks	17*

*Electrodes	18*

*Baseline	19*

*ERN Time Window	20*

*Amplitude Measurement	20*

##### Supplementary Table 1: Overview X² Tests

| **Predictor** | **Variable** | **Model** | **df** | **𝜒/*t*** | ***p*** |  | ***V/R²*** | **CI95** |
| --- | --- | --- | --- | --- | --- | --- | --- | --- |
| Year | Experimental Task | Multinominal | 25 | 49.65 | .002 |  | 0.10 | 0.12 - 0.17 |
|  | Amplifier Type | Multinominal | 25 | 93.32 | ≤ .001 | * | 0.13 | 0.14 - 0.23 |
|  | Electrode Type | Multinominal | 10 | 66.78 | ≤ .001 | * | 0.16 | 0.15 - 0.25 |
|  | Nr. Recorded Channels | Continous | 199 | 4.06 | .0001 |  | 0.08 | 0.14 - 0.41 |
|  | Nr. Recorded Channels | Multinominal | 15 | 24.24 | .061 |  | 0.05 | 0.07 - 0.11 |
|  | EOG Recording | Multinominal | 5 | 9.16 | .103 |  | 0.04 | 0.04 - 0.10 |
|  | Preprocessing Sortware | Multinominal | 25 | 107.18 | ≤ .001 | * | 0.19 | 0.16 - 0.26 |
|  | Sampling Rate | Multinominal | 25 | 70.13 | ≤ .001 | * | 0.13 | 0.08 - 0.23 |
|  | Sample Size (All) | Continous | 200 | 8.10 | .0000 | * | 0.25 | 0.38 - 0.62 |
|  | Sample Size (All) | Multinominal | 20 | 52.20 | ≤ .001 |  | 0.08 | 0.07 - 0.17 |
|  | Sample Size (Within) | Continous | 66 | 5.75 | .0000 | * | 0.33 | 0.38 - 0.78 |
|  | Sample Size (Within) | Multinominal | 20 | 36.56 | .013 |  | 0.22 | 0.21 - 0.37 |
|  | Sample Size (Between) | Continous | 132 | 6.39 | .0000 | * | 0.24 | 0.34 - 0.64 |
|  | Sample Size (Between) | Multinominal | 20 | 43.11 | .002 |  | 0.11 | 0.11 - 0.17 |
|  | Reference | Multinominal | 25 | 40.30 | .027 |  | 0.08 | 0.10 - 0.19 |
|  | Low Pass Filter | Continous | 194 | 3.59 | .0004 |  | 0.06 | 0.11 - 0.39 |
|  | Low Pass Filter | Multinominal | 15 | 34.20 | .003 |  | 0.07 | 0.06 - 0.15 |
|  | High Pass Filter | Continous | 180 | -0.30 | .7616 |  | 0.00 | -0.17 - 0.12 |
|  | High Pass Filter | Multinominal | 15 | 21.37 | .125 |  | 0.05 | 0.06 - 0.14 |
|  | Artifacts Detection | Multinominal | 15 | 14.07 | .520 |  | 0.03 | 0.05 - 0.09 |
|  | Occular Correction | Multinominal | 15 | 77.75 | ≤ .001 | * | 0.18 | 0.15 - 0.25 |
|  | Channel Correction | Logistic | 5 | 45.88 | ≤ .001 | * | 0.24 | 0.16 - 0.36 |
|  | Central Electrode | Multinominal | 20 | 34.46 | .023 |  | 0.09 | 0.09 - 0.16 |
|  | Number Electrodes | Multinominal | 15 | 17.35 | .299 |  | 0.04 | 0.05 - 0.09 |
|  | Pooling Electrodes | Multinominal | 15 | 34.72 | .003 |  | 0.08 | 0.07 - 0.15 |
|  | Baseline Length | Continous | 182 | 2.75 | .0066 |  | 0.04 | 0.06 - 0.34 |
|  | Baseline Length | Multinominal | 20 | 39.36 | .006 |  | 0.11 | 0.10 - 0.21 |
|  | Baseline Center | Continous | 182 | -3.33 | .0010 |  | 0.06 | -0.38 - -0.10 |
|  | Baseline Center | Multinominal | 15 | 38.14 | .001 | * | 0.10 | 0.11 - 0.18 |
|  | Time Window Length | Continous | 185 | -4.09 | .0001 |  | 0.08 | -0.43 - -0.15 |
|  | Time Window Length | Multinominal | 20 | 44.61 | .001 |  | 0.09 | 0.08 - 0.19 |
|  | Time Window Center | Continous | 185 | -5.77 | .0000 | * | 0.15 | -0.52 - -0.26 |
|  | Time Window Center | Multinominal | 15 | 39.30 | .001 |  | 0.08 | 0.08 - 0.15 |
|  | Amplitude Measurement | Multinominal | 30 | 68.94 | ≤ .001 | * | 0.12 | 0.10 - 0.17 |
| Experimental | Reference | Multinominal | 25 | 42.36 | .016 |  | 0.09 | 0.08 - 0.20 |
| Task | Low Pass Filter | Multinominal | 15 | 26.35 | .035 |  | 0.06 | 0.05 - 0.16 |
|  | High Pass Filter | Multinominal | 15 | 15.08 | .446 |  | 0.03 | 0.04 - 0.08 |
|  | Artifacts Detection | Multinominal | 15 | 35.78 | .002 |  | 0.07 | 0.07 - 0.12 |
|  | Occular Correction | Multinominal | 15 | 18.83 | .222 |  | 0.04 | 0.05 - 0.08 |
|  | Channel Correction | Logistic | 5 | 7.79 | .168 |  | 0.04 | 0.02 - 0.14 |
|  | Central Electrode | Multinominal | 20 | 19.99 | .458 |  | 0.05 | 0.06 - 0.12 |
|  | Number Electrodes | Multinominal | 15 | 19.44 | .194 |  | 0.04 | 0.04 - 0.10 |
|  | Pooling Electrodes | Multinominal | 15 | 21.49 | .122 |  | 0.05 | 0.03 - 0.10 |
|  | Baseline Length | Multinominal | 20 | 23.52 | .264 |  | 0.06 | 0.05 - 0.15 |
|  | Baseline Center | Multinominal | 15 | 24.77 | .053 |  | 0.07 | 0.06 - 0.15 |
|  | Time Window Length | Multinominal | 20 | 36.38 | .014 |  | 0.07 | 0.06 - 0.14 |
|  | Time Window Center | Multinominal | 15 | 19.73 | .182 |  | 0.04 | 0.05 - 0.15 |
|  | Amplitude Measurement | Multinominal | 30 | 55.13 | .003 |  | 0.10 | 0.10 - 0.17 |
| Amplifier Type | Reference | Multinominal | 25 | 75.36 | ≤ .001 | * | 0.15 | 0.14 - 0.22 |
|  | Low Pass Filter | Multinominal | 15 | 46.48 | ≤ .001 | * | 0.10 | 0.09 - 0.17 |
|  | High Pass Filter | Multinominal | 15 | 29.20 | .015 |  | 0.06 | 0.07 - 0.13 |
|  | Artifacts Detection | Multinominal | 15 | 30.37 | .011 |  | 0.06 | 0.09 - 0.13 |
|  | Occular Correction | Multinominal | 15 | 44.47 | ≤ .001 | * | 0.10 | 0.10 - 0.15 |
|  | Channel Correction | Logistic | 5 | 46.42 | ≤ .001 | * | 0.24 | 0.18 - 0.36 |
|  | Central Electrode | Multinominal | 20 | 28.50 | .098 |  | 0.08 | 0.09 - 0.14 |
|  | Number Electrodes | Multinominal | 15 | 35.18 | .002 |  | 0.08 | 0.06 - 0.17 |
|  | Pooling Electrodes | Multinominal | 15 | 55.86 | ≤ .001 | * | 0.13 | 0.07 - 0.19 |
|  | Baseline Length | Multinominal | 20 | 40.12 | .005 |  | 0.11 | 0.11 - 0.19 |
|  | Baseline Center | Multinominal | 15 | 55.70 | ≤ .001 | * | 0.15 | 0.15 - 0.26 |
|  | Time Window Length | Multinominal | 20 | 41.88 | .003 |  | 0.08 | 0.09 - 0.17 |
|  | Time Window Center | Multinominal | 15 | 35.90 | .002 |  | 0.07 | 0.09 - 0.17 |
|  | Amplitude Measurement | Multinominal | 30 | 65.16 | ≤ .001 | * | 0.11 | 0.11 - 0.20 |
| Electrode | Reference | Multinominal | 10 | 35.82 | ≤ .001 |  | 0.07 | 0.07 - 0.12 |
| Type | Low Pass Filter | Multinominal | 6 | 19.63 | .003 |  | 0.04 | 0.03 - 0.07 |
|  | High Pass Filter | Multinominal | 6 | 17.75 | .007 |  | 0.04 | 0.02 - 0.09 |
|  | Artifacts Detection | Multinominal | 6 | 23.59 | .001 |  | 0.05 | 0.05 - 0.14 |
|  | Occular Correction | Multinominal | 6 | 16.49 | .011 |  | 0.04 | 0.02 - 0.07 |
|  | Channel Correction | Logistic | 2 | 23.12 | ≤ .001 | * | 0.12 | 0.08 - 0.21 |
|  | Central Electrode | Multinominal | 8 | 7.47 | .487 |  | 0.02 | 0.02 - 0.07 |
|  | Number Electrodes | Multinominal | 6 | 13.02 | .043 |  | 0.03 | 0.03 - 0.05 |
|  | Pooling Electrodes | Multinominal | 6 | 11.76 | .067 |  | 0.03 | 0.02 - 0.06 |
|  | Baseline Length | Multinominal | 8 | 24.92 | .002 |  | 0.07 | 0.05 - 0.11 |
|  | Baseline Center | Multinominal | 6 | 26.93 | ≤ .001 |  | 0.07 | 0.06 - 0.13 |
|  | Time Window Length | Multinominal | 8 | 19.95 | .011 |  | 0.04 | 0.03 - 0.10 |
|  | Time Window Center | Multinominal | 6 | 19.03 | .004 |  | 0.04 | 0.03 - 0.09 |
|  | Amplitude Measurement | Multinominal | 12 | 27.25 | .007 |  | 0.05 | 0.04 - 0.09 |
| Nr. Recorded | Reference | Multinominal | 15 | 75.85 | ≤ .001 | * | 0.15 | 0.12 - 0.21 |
| Channels | Low Pass Filter | Continous | 193 | 0.02 | .9874 |  | 0.00 | -0.14 - 0.14 |
|  | Low Pass Filter | Multinominal | 9 | 25.54 | .002 |  | 0.05 | 0.04 - 0.13 |
|  | High Pass Filter | Continous | 179 | 2.68 | .0081 |  | 0.04 | 0.05 - 0.34 |
|  | High Pass Filter | Multinominal | 9 | 19.79 | .019 |  | 0.04 | 0.04 - 0.10 |
|  | Artifacts Detection | Multinominal | 9 | 9.00 | .437 |  | 0.02 | 0.01 - 0.07 |
|  | Occular Correction | Multinominal | 9 | 14.87 | .095 |  | 0.03 | 0.03 - 0.10 |
|  | Channel Correction | Logistic | 3 | 18.25 | ≤ .001 | * | 0.10 | 0.05 - 0.20 |
|  | Central Electrode | Multinominal | 12 | 10.35 | .586 |  | 0.03 | 0.03 - 0.11 |
|  | Number Electrodes | Multinominal | 9 | 26.65 | .002 |  | 0.06 | 0.05 - 0.12 |
|  | Pooling Electrodes | Multinominal | 9 | 37.80 | ≤ .001 |  | 0.09 | 0.07 - 0.13 |
|  | Baseline Length | Continous | 182 | 3.99 | .0001 |  | 0.08 | 0.14 - 0.42 |
|  | Baseline Length | Multinominal | 12 | 24.13 | .020 |  | 0.07 | 0.05 - 0.13 |
|  | Baseline Center | Continous | 182 | -2.60 | .0101 |  | 0.04 | -0.33 - -0.05 |
|  | Baseline Center | Multinominal | 9 | 27.71 | .001 |  | 0.07 | 0.04 - 0.12 |
|  | Time Window Length | Continous | 184 | -3.79 | .0002 |  | 0.07 | -0.41 - -0.13 |
|  | Time Window Length | Multinominal | 12 | 16.72 | .160 |  | 0.03 | 0.04 - 0.11 |
|  | Time Window Center | Continous | 184 | -1.56 | .1207 |  | 0.01 | -0.26 - 0.03 |
|  | Time Window Center | Multinominal | 9 | 7.69 | .566 |  | 0.02 | 0.02 - 0.09 |
|  | Amplitude Measurement | Multinominal | 18 | 27.15 | .076 |  | 0.05 | 0.03 - 0.10 |
| EOG | Reference | Multinominal | 5 | 18.22 | .003 |  | 0.04 | 0.02 - 0.06 |
| Recording | Low Pass Filter | Multinominal | 3 | 0.36 | .949 |  | 0.00 | ≤ 0.01 - 0.03 |
|  | High Pass Filter | Multinominal | 3 | 5.24 | .155 |  | 0.01 | 0.01 - 0.03 |
|  | Artifacts Detection | Multinominal | 3 | 2.90 | .407 |  | 0.01 | ≤ 0.01 - 0.03 |
|  | Occular Correction | Multinominal | 3 | 9.82 | .020 |  | 0.02 | 0.01 - 0.07 |
|  | Channel Correction | Logistic | 1 | 13.42 | ≤ .001 |  | 0.07 | 0.02 - 0.17 |
|  | Central Electrode | Multinominal | 4 | 6.36 | .174 |  | 0.02 | 0.01 - 0.04 |
|  | Number Electrodes | Multinominal | 3 | 6.51 | .089 |  | 0.01 | ≤ 0.01 - 0.04 |
|  | Pooling Electrodes | Multinominal | 3 | 8.98 | .030 |  | 0.02 | ≤ 0.01 - 0.05 |
|  | Baseline Length | Multinominal | 4 | 2.65 | .617 |  | 0.01 | ≤ 0.01 - 0.04 |
|  | Baseline Center | Multinominal | 3 | 4.86 | .182 |  | 0.01 | ≤ 0.01 - 0.04 |
|  | Time Window Length | Multinominal | 4 | 8.88 | .064 |  | 0.02 | ≤ 0.01 - 0.05 |
|  | Time Window Center | Multinominal | 3 | 6.19 | .103 |  | 0.01 | 0.01 - 0.04 |
|  | Amplitude Measurement | Multinominal | 6 | 15.28 | .018 |  | 0.03 | 0.02 - 0.05 |
| Preprocessing | Reference | Multinominal | 25 | 69.36 | ≤ .001 | * | 0.14 | 0.14 - 0.20 |
| Software | Low Pass Filter | Multinominal | 15 | 38.25 | .001 |  | 0.08 | 0.07 - 0.16 |
|  | High Pass Filter | Multinominal | 15 | 23.21 | .080 |  | 0.05 | 0.06 - 0.12 |
|  | Artifacts Detection | Multinominal | 15 | 32.06 | .006 |  | 0.06 | 0.05 - 0.14 |
|  | Occular Correction | Multinominal | 15 | 134.56 | ≤ .001 | * | 0.31 | 0.27 - 0.40 |
|  | Channel Correction | Logistic | 5 | 51.77 | ≤ .001 | * | 0.27 | 0.17 - 0.45 |
|  | Central Electrode | Multinominal | 20 | 12.79 | .886 |  | 0.03 | 0.04 - 0.08 |
|  | Number Electrodes | Multinominal | 15 | 20.53 | .152 |  | 0.05 | 0.05 - 0.12 |
|  | Pooling Electrodes | Multinominal | 15 | 31.31 | .008 |  | 0.07 | 0.06 - 0.13 |
|  | Baseline Length | Multinominal | 20 | 32.19 | .041 |  | 0.09 | 0.09 - 0.17 |
|  | Baseline Center | Multinominal | 15 | 35.38 | .002 |  | 0.10 | 0.10 - 0.16 |
|  | Time Window Length | Multinominal | 20 | 45.91 | .001 |  | 0.09 | 0.09 - 0.16 |
|  | Time Window Center | Multinominal | 15 | 34.27 | .003 |  | 0.07 | 0.06 - 0.13 |
|  | Amplitude Measurement | Multinominal | 30 | 42.01 | .071 |  | 0.07 | 0.07 - 0.16 |
| Sampling Rate | Reference | Multinominal | 25 | 21.99 | .636 |  | 0.04 | 0.04 - 0.09 |
|  | Low Pass Filter | Multinominal | 15 | 21.41 | .124 |  | 0.04 | 0.05 - 0.10 |
|  | High Pass Filter | Multinominal | 15 | 16.79 | .331 |  | 0.04 | 0.04 - 0.09 |
|  | Artifacts Detection | Multinominal | 15 | 31.33 | .008 |  | 0.06 | 0.06 - 0.14 |
|  | Occular Correction | Multinominal | 15 | 28.79 | .017 |  | 0.07 | 0.05 - 0.14 |
|  | Channel Correction | Logistic | 5 | 15.42 | .009 |  | 0.08 | 0.04 - 0.19 |
|  | Central Electrode | Multinominal | 20 | 20.13 | .450 |  | 0.05 | 0.05 - 0.13 |
|  | Number Electrodes | Multinominal | 15 | 13.03 | .600 |  | 0.03 | 0.02 - 0.07 |
|  | Pooling Electrodes | Multinominal | 15 | 15.83 | .393 |  | 0.04 | 0.02 - 0.09 |
|  | Baseline Length | Multinominal | 20 | 35.97 | .015 |  | 0.10 | 0.06 - 0.19 |
|  | Baseline Center | Multinominal | 15 | 31.87 | .007 |  | 0.09 | 0.08 - 0.15 |
|  | Time Window Length | Multinominal | 20 | 33.76 | .028 |  | 0.07 | 0.05 - 0.13 |
|  | Time Window Center | Multinominal | 15 | 19.50 | .192 |  | 0.04 | 0.04 - 0.09 |
|  | Amplitude Measurement | Multinominal | 30 | 20.16 | .912 |  | 0.04 | 0.04 - 0.09 |
| Sample Size (All) | Artifacts Detection | Multinominal | 12 | 15.99 | .192 |  | 0.03 | 0.04 - 0.07 |
|  | Occular Correction | Multinominal | 12 | 29.45 | .003 |  | 0.07 | 0.06 - 0.13 |
|  | Channel Correction | Logistic | 4 | 19.65 | .001 | * | 0.10 | 0.04 - 0.24 |
| Sample Size | Artifacts Detection | Multinominal | 12 | 15.99 | .192 |  | 0.03 | 0.04 - 0.07 |
| (Within) | Occular Correction | Multinominal | 12 | 29.45 | .003 |  | 0.07 | 0.06 - 0.13 |
|  | Channel Correction | Logistic | 4 | 19.65 | .001 | * | 0.10 | 0.04 - 0.24 |
| Sample Size | Artifacts Detection | Multinominal | 12 | 15.99 | .192 |  | 0.03 | 0.04 - 0.07 |
| (Between) | Occular Correction | Multinominal | 12 | 29.45 | .003 |  | 0.07 | 0.06 - 0.13 |
|  | Channel Correction | Logistic | 4 | 19.65 | .001 | * | 0.10 | 0.04 - 0.24 |

*Note.* Reporting of all pre-defined combinations of choices. * indicates p < 0.001 & VR² > 0.1

##### Supplementary Table 2: Remaining Entropy after Controlling for several Variables

| **Target Variable** | **Variables Controlled for** | **Original Entropy** | **Remaining Entropy** |
| --- | --- | --- | --- |
| Low Pass Filter | Amplifier Type | 0.80 | 0.80 |
| Occular Correction | Preprocessing Software | 0.78 | 0.61 |
|  | Year | 0.78 | 0.72 |
|  | Amplifier Type | 0.78 | 0.77 |
|  | Preprocessing Software & Year | 0.75 | 0.74 |
|  | Preprocessing Software & Year & Amplifier Type | 0.78 | 0.86 |
| Channel Correction | Preprocessing Software | 0.70 | 0.52 |
|  | Sample Size | 0.68 | 0.61 |
|  | Nr. Recorded Channels | 0.71 | 0.67 |
|  | Year | 0.81 | 0.71 |
|  | Electrode Type | 0.75 | 0.72 |
|  | Amplifier Type | 0.84 | 0.76 |
|  | Preprocessing Software & Sample Size | 0.88 | 0.71 |
|  | Preprocessing Software & Sample Size & Nr. Recorded Channels | 0.95 | 0.81 |
|  | Preprocessing Software & Sample Size & Nr. Recorded Channels & Year | 0.99 | 0.93 |
|  | Preprocessing Software & Sample Size & Nr. Recorded Channels & Year & Electrode Type | 0.99 | 0.95 |
|  | Preprocessing Software & Sample Size & Nr. Recorded Channels & Year & Electrode Type & Amplifier Type | 0.97 | 0.93 |
| Pooling Electrodes | Amplifier Type | 0.78 | 0.81 |
| Reference | Nr. Recorded Channels | 0.68 | 0.62 |
|  | Amplifier Type | 0.68 | 0.70 |
|  | Preprocessing Software | 0.68 | 0.70 |
|  | Nr. Recorded Channels & Amplifier Type | 0.70 | 0.76 |
|  | Nr. Recorded Channels & Amplifier Type & Preprocessing Software | 0.70 | 0.80 |
| Amplitude Measurement | Amplifier Type | 0.74 | 0.77 |
|  | Year | 0.74 | 0.78 |
|  | Amplifier Type & Year | 0.76 | 0.84 |
| Baseline Center | Amplifier Type | 0.76 | 0.75 |
|  | Year | 0.76 | 0.77 |
|  | Amplifier Type & Year | 0.78 | 0.83 |
| Time Window Center | Year | 0.91 | 0.90 |

*Note.* Total Entropy is the entropy of the target variable (these changes depending on the studies that were present in the certain combinations. Remaining entropy is the entropy after controlling for all of listed variables.

##### Supplementary Table 3: Inter Rater Reliability.

| **Variable** | **Kappa** | ***z*** | ***p*** |
| --- | --- | --- | --- |
| Preregistration | 1.00 | 6.63 | ≤ .001 |
| Within/Between Design | 0.95 | 6.32 | ≤ .001 |
| Experimental Task | 0.84 | 9.07 | ≤ .001 |
| Amplifier Type | 0.97 | 13.28 | ≤ .001 |
| Number Recorded Channels | 0.80 | 17.17 | ≤ .001 |
| EOG Recording | 0.82 | 5.56 | ≤ .001 |
| Preprocessing Software Type | 0.93 | 10.47 | ≤ .001 |
| Sampling Rate | 1.00 | 9.12 | ≤ .001 |
| Sample Size | 0.90 | 28.21 | ≤ .001 |
| Reference | 0.93 | 10.13 | ≤ .001 |
| Low Pass Filter | 0.88 | 14.20 | ≤ .001 |
| High Pass Filter | 0.89 | 14.61 | ≤ .001 |
| Artifacts Detection | 0.73 | 7.96 | ≤ .001 |
| Occular Correction | 0.82 | 7.89 | ≤ .001 |
| Channel Correction | 1.00 | 6.63 | ≤ .001 |
| Fronto (Central) Electrodes | 0.92 | 15.00 | ≤ .001 |
| Central Electrode | 0.79 | 7.88 | ≤ .001 |
| Number Relevant Electrodes | 0.93 | 11.49 | ≤ .001 |
| Pooling Electrodes | 0.81 | 7.17 | ≤ .001 |
| Baseline Length | 1.00 | 6.71 | ≤ .001 |
| Baseline Center | 1.00 | 13.13 | ≤ .001 |
| Time Window Length | 1.00 | 15.34 | ≤ .001 |
| Time Window Center | 1.00 | 18.48 | ≤ .001 |
| Amplitude Measurement | 0.87 | 10.06 | ≤ .001 |

*Note.* *N* = 44 studies

##### Supplementary Table 4. List of all coded Variables

| **Descriptives** |  |
| --- | --- |
| Doi, PMID, PsycID, FileName |  |
| Year |  |
| First Author (Name + Initials) |  |
| Last Author (Name + Initials) |  |
| Country |  |
| **Study Setup** |  |
| Preregistered | Yes, No/Not Reported |
| Sample Age (Mean, SD, Range) |  |
| Sample Size |  |
| Task | Flanker, Stroop, Go/No-Go, Simon, other |
| Task Detail | *More details if relevant* |
| Was psychopathology measured? | Yes/No |
| Was personality measured? | Yes/No |
| Within-Between Design | Within/Between |
| Electrode Type | Active/Passive |
| Amplifier | Biosemi, BrainProducts, Geodesic, (Neuro)Scan, Other, Not Reported |
| Amplifier Detail | *More specific than above (relevant for other)* |
| Software Processing | BrainVision Analyzer, EEGlab, (Neuro)Scan, BESA, Other, Not Reported |
| Software Processing Detail | *More specific than above (relevant for other)* |
| Number of recorded Channels |  |
| EOG Channels | EOG only, VEOG only, both, none |
| Recording Reference | one mastoid, one earlobe, both mastoids, both earlobes, central midline, face (chin, forehead, nose), other, CMS/DRL, Not Reported , Other |
| Sampling Rate Recording |  |
| Online high-pass filter cut-off (Hz) |  |
| Online low-pass filter cut-off (Hz) |  |
| **Preprocessing** |  |
| Downsampling | Yes/No |
| Sampling Rate Analysis | *(from Sampling Rate Recording & Downsampling, rounded last digit)* |
| Offline high-pass filter cut-off |  |
| Offline low-pass filter cut-off (Hz) |  |
| Notch Filter | Yes/No |
| Order Offline Filter |  |
| Final Low Pass | *(from online/offline filter)* |
| Final High Pass | *(from online/offline filter)* |
| Offline Reference | Mastoids, earlobes, average, one mastoid, CSD, Not Applied |
| Order Reference |  |
| Final Reference | *(from Online/Offline Reference)*  Mastoids, Earlobes, Average, CSD, Others, Not Reported |
| Artifact Detection | Visual, Combination Including Manual, Combination only Automatic, Not Applied |
| Artifact Detection Detail | *More details if provided, e.g. cutoffs* |
| Order Artifacts Detection |  |
| Scanned for Bad Channels | Yes, No |
| Bad Channels Detail | *More details if provided* |
| Order Bad channels |  |
| Ocular Correction | Regression, ICA/BSS, Only general Artifacts Det., Not Applied |
| Ocular Correction Detail | *More details if provided* |
| Order Ocular Correction |  |
| Any Correction method | Yes/No *from Artifacts Det. and Ocular Cor.* |
| **ERN Quantification** |  |
| Reference point for Epoching, Baseline and ERN Time window | Error, EMG, Stimulus, Other (e.g. calibrated) |
| Epoching (Start, End, Length) |  |
| Order Epoching |  |
| Baseline Time Window (Start, End, Length, Center) |  |
| ERN Time Window (Start, End, Length, Center) |  |
| TimeAroundPeak(Total) | *(in case there was mean around peak)* |
| Timepoint Averaging | Before Quantification, Earlier |
| Time window end of Baseline to start of ERN Window | *(from Baseline and ERN window)* |
| Difference Waves? | Separate, Difference, Transformation |
| ERNDefinition | Mean, Peak, Component Difference (measure-to-measure), Mean around found Peak, Multiple, Not Reported |
| Electrodes in Stats in Total |  |
| Fronto(central) Electrodes |  |
| Number frontal Electrodes |  |
| Central Electrode(s) | Cz, FCz, Fz, FCz/Cz, Fz/FCz, FCz |
| Merging Electrodes (per Cluster) | Yes, No, Not Applicable |
| **Exclusions** |  |
| Reaction Time Limits (Low, High) | *Not from Task but which were considered in EEG analysis* |
| Minimum Trials |  |
| Any outliers | Yes/No |
| Outliers Detail | *More details if provided* |
| Other ERPs examined | Yes/No |
| Other ERPs Details |  |

##### Supplementary Figure 1: Distribution of how continuous variables were grouped

Supplementary Figures 2+: Mosaic Plots of shared occurrence of multiple choices.
Distribution of choices for different combinations presented as a mosaic plot. The color blue indicates the most frequent category of the x axis (across all levels of the y axis). Similarly, the row’s height on the y axis indicates the frequency of the categories on the y axis (across all levels of the x axis). The width of each rectangle shows the relative distribution of one category on the y axis across the levels of the y axis. Bold colors and +/- indicate combinations significantly over- or under-represented (as defined by a $X^{2}$ test). Only cells with at least 5 occurrences are shown. Plots surrounded by a frame were significant across our criteria (*p* < 0.001, *V/R²* > 0.1).

Supplementary Figure 2:

Supplementary Figure 3:

Supplementary Figure 4:

Supplementary Figure 5:

Supplementary Figure 6:

Supplementary Figure 7:

Supplementary Figure 8:

Supplementary Figure 9:

Supplementary Figure 10:

Supplementary Figure 11:

Supplementary Figure 12:

Supplementary Figure 13:

Supplementary Figure 14:

Supplementary Figure 15:

Supplementary Figure 16:

Supplementary Figure 17:
